# MALAT1–miR-20b-5p–P2RX7 Axis Regulates *Mycobacterium bovis*-Induced THP-1 Pyroptosis

**DOI:** 10.3390/vetsci13060545

**Published:** 2026-05-31

**Authors:** Tian Tian, Xiaonan Wang, Yanan Zhu, Qi Wang, Wei Zheng, Kun Shi, Rui Du

**Affiliations:** 1 College of Veterinary Medicine, Jilin Agricultural University, Changchun 130118, China; 2 College of Chinese Medicinal Materials, Jilin Agricultural University, Changchun 130118, China; 3 College of Agriculture, Yanbian University, Yanji 133002, China

**Keywords:** MALAT1, miR-20b-5p, *Mycobacterium bovis*, non-coding RNA, P2RX7, pyroptosis, THP-1 cells, zoonotic tuberculosis

## Abstract

Zoonotic tuberculosis (zoonotic TB) caused by *Mycobacterium bovis* (*M. bovis*) accounts for up to 10% of human tuberculosis cases in some regions, but the underlying pathogenic mechanisms remain incompletely understood, especially those involved in cellular pyroptosis. This study aimed to explore the regulatory roles of non-coding RNA (ncRNA) in the pyroptosis of human monocytic THP-1 cells induced by *M. bovis* infection. An in vitro pyroptosis model was established by infecting THP-1 cells with *M. bovis*, followed by whole-transcriptome sequencing to identify differentially expressed messenger RNA (mRNA), long non-coding RNA (lncRNA), microRNA (miRNA), and circular RNA (circRNA). Bioinformatics analysis was performed to construct an lncRNA–miRNA–mRNA regulatory network associated with infection-induced pyroptosis; in addition, overexpression, knockdown, and dual-luciferase reporter assays and quantitative PCR were conducted to verify the interactions and functions of metastasis-associated lung adenocarcinoma transcript 1 (MALAT1), miR-20b-5p, and purinergic receptor P2X7 (P2RX7). Transcriptome analysis detected 741 mRNAs, 1049 lncRNAs, 25 circRNAs, and 40 miRNAs with significant differential expression in infected THP-1 cells. Specifically, MALAT1 and P2RX7 were upregulated, while miR-20b-5p was downregulated after infection. Knockdown of MALAT1 or P2RX7 and overexpression of miR-20b-5p relieved *M. bovis*-induced pyroptosis in THP-1 cells. Mechanistically, MALAT1 targeted miR-20b-5p, which directly targeted P2RX7, and overexpression of miR-20b-5p partially reversed P2RX7 upregulation mediated by MALAT1 overexpression. This study provides a transcriptomic characterization of *M. bovis*-induced pyroptosis in THP-1 cells and supports the MALAT1–miR-20b-5p–P2RX7 axis as a potential regulatory mechanism involved in this process, offering initial molecular insights into the pathogenesis of zoonotic TB.

## Simple Summary

Zoonotic tuberculosis caused by *Mycobacterium bovis* is a global public health problem, yet the mechanisms governing this infection—including host immune responses and cell death pathways—remain poorly understood. Pyroptosis is a pro-inflammatory form of cell death that mediates host defense against mycobacterial infection. This study explores non-coding RNA regulatory networks involved in *Mycobacterium bovis*-induced pyroptosis in human monocytic cells. These findings provide initial molecular insights into the host immune response to *Mycobacterium bovis* infection and identify a regulatory axis that merits further investigation. Additional studies using primary macrophages, animal models, and clinical specimens are needed to assess the potential relevance of this axis for diagnosis and treatment.

## 1. Introduction

Tuberculosis (TB) remains a leading cause of death from a single infectious agent, surpassing HIV/AIDS. According to the WHO Global Tuberculosis Report 2024, an estimated 10.8 million new TB cases and 1.25 million deaths occurred in 2023 [1]. Among these cases, zoonotic TB caused by *M. bovis* continues to be a neglected burden. Although precise figures are hampered by diagnostic limitations, the WHO estimated that approximately 140,000 new cases and 11,400 deaths were attributable to *M. bovis* infections globally in 2019, with the true burden likely substantially underestimated [2,3]. *M. bovis* primarily affects cattle (including dairy cows, yellow cattle, buffalo, and yaks) but can also infect other animals and humans, and has a wide geographical distribution [4,5]. Disparities in resources, surveillance, and diagnostics have created a notable research gap between zoonotic and human TB, and an in-depth understanding of the molecular mechanisms underlying *M. bovis* infection is urgently needed.

Pyroptosis is a key component of innate immunity, characterized by inflammasome-triggered caspase activation, particularly caspase-1, leading to gasdermin D (GSDMD) cleavage, pore formation in cell membranes, and the release of interleukin-1 beta (IL-1β) and interleukin-18 (IL-18). Extensive research has linked pyroptosis to *Mycobacterium tuberculosis* (*M. tuberculosis*) infection; for instance, *M. tuberculosis* activates the NLR family pyrin domain-containing 3 (NLRP3) inflammasome via the ESX-1 system and K^+^ efflux, and the EST12 protein recruits the deubiquitinase UCHL5 to stabilize NLRP3, thereby promoting pyroptosis [6,7]. Conversely, *M. tuberculosis* also employs virulence factors such as PknF to suppress inflammasome activation [8,9]. Given the high genomic homology between *M. tuberculosis* and *M. bovis* [10], along with the conserved nature of the inflammasome–pyroptosis pathway, findings from *M. tuberculosis* provide a valuable foundation for investigating *M. bovis* infection. Importantly, emerging evidence directly demonstrates that *M. bovis* activates a conserved inflammasome–pyroptosis axis in infected macrophages. Infection of bovine macrophages with the *M. bovis* AN5 strain induces NLRP3 inflammasome activation, ASC oligomerization, caspase-1 cleavage, GSDMD processing, and subsequent IL-1β release and pyroptotic cell death. Moreover, GSDMD-positive macrophages have been detected in lymph node tissues from naturally *M. bovis*-infected cattle [11]. The attenuated BCG strain also triggers endoplasmic reticulum stress and activates the NLRP3 inflammasome via the PERK/ATF4/CHOP pathway in THP-1 macrophages [12]. Despite these advances, the specific regulatory networks governing *M. bovis*-induced pyroptosis—particularly those involving ncRNAs—remain largely uncharacterized.

Whole-transcriptome sequencing identifies protein-coding mRNAs and ncRNAs, including miRNAs, lncRNAs, and circRNAs. ncRNAs regulate pyroptosis through diverse mechanisms. One well-characterized mechanism is the competing endogenous RNA (ceRNA) pathway, in which lncRNAs and circRNAs act as molecular sponges that bind to miRNAs, thereby relieving miRNA-induced repression of pyroptosis-related target genes. A recent study demonstrated that, in *M. tuberculosis*-infected macrophages, MALAT1 functions through the ceRNA mechanism by sponging miR-125b to derepress TLR4, thereby promoting NLRP3-mediated pyroptosis [13]. Despite these advances in *M. tuberculosis* research, whether and how ncRNAs regulate *M. bovis*-induced pyroptosis in THP-1 macrophages remains poorly understood. For this study, we established an *M. bovis*-induced THP-1 pyroptosis model and performed whole-transcriptome sequencing to identify differentially expressed RNAs. We constructed a ceRNA regulatory network and identified significant upregulation of MALAT1 and P2RX7, along with downregulation of miR-20b-5p. Functional assays demonstrated that silencing MALAT1 or P2RX7, or overexpressing miR-20b-5p, alleviated pyroptosis. Mechanistically, MALAT1 functions as a ceRNA by sponging miR-20b-5p to regulate P2RX7 expression. These findings suggest a novel MALAT1–miR-20b-5p–P2RX7 axis that may control *M. bovis*-induced pyroptosis, providing preliminary molecular insight into the host response and identifying a regulatory axis that warrants further validation for the prevention and control of zoonotic TB.

## 2. Materials and Methods

### 2.1. Cell Culture

Human monocyte cell line THP-1 cells were purchased from the cell bank of the Typical Culture Preservation Committee of the Chinese Academy of Sciences. Cell authentication was performed by the supplier using short tandem repeat (STR) profiling. Upon arrival, cells were expanded and cryopreserved. All experiments were conducted using cells at passages 5–20 after thawing to minimize phenotypic drift. Routine mycoplasma testing was performed monthly using a PCR-based detection kit (Beyotime, Shanghai, China), and all cells used in this study tested negative. Cells were cultured in Roswell Park Memorial Institute-1640 (RPMI-1640) medium (Gibco, Invitrogen, Grand Island, NY, USA) supplemented with 10% fetal bovine serum (Royacel^®^ SERUM, Lanzhou Rongye Biotechnology Co., Ltd., Lanzhou, China) and 1% penicillin–streptomycin (Solarbio, Beijing, China). Cells were maintained in a constant temperature incubator at 37 °C with 5% CO_2_. To induce the transformation of monocytes into macrophages, THP-1 cells were incubated with 100 ng/mL phorbol 12–myristate 13–acetate (PMA; Sigma-Aldrich, Darmstadt, Germany) for 24 h prior to subsequent experiments. Following PMA treatment, cells were washed twice with warm phosphate-buffered saline (PBS) and rested in fresh medium without antibiotics, supplemented with 10% FBS, for an additional 24 h prior to infection.

### 2.2. Infection of THP-1 Cells with M. bovis

The *M. bovis* strain used in this study was isolated and preserved by our laboratory. The strain is virulent and was cultured in Middlebrook 7H9 broth (BD, Franklin Lakes, NJ, USA) supplemented with 10% OADC enrichment (BD, Franklin Lakes, NJ, USA) and 0.05% Tween 80 (Sigma-Aldrich, Germany) at 37 °C to mid-log phase (OD_600_ ≈ 0.6–0.8). Bacterial clumps were dispersed via repeated pipetting with a sterile syringe (>50 times) to obtain a single-cell suspension. The bacterial concentration was determined by measuring OD_600_ and confirmed via colony-forming unit (CFU) plating on Middlebrook 7H10 agar (BD, Franklin Lakes, NJ, USA). All live *M. bovis* manipulations were performed in a biosafety level 3 (BSL-3) facility. Prior to infection, penicillin–streptomycin was removed from the cell culture medium.

Two independent experimental setups were performed to evaluate the effects of different infection conditions. For the multiplicity of infection (MOI) experiment, THP-1 cells were co-cultured with *M. bovis* at MOIs of 1, 10, and 100. The time of bacterial addition was defined as 0 h post-infection (hpi). After 2 h co-incubation at 37 °C, the culture medium containing bacteria was discarded, and cells were washed three times with pre-warmed PBS. Cells were then cultured in RPMI-1640 medium supplemented with gentamicin (50 μg/mL, Solarbio, China) and 2% fetal bovine serum. After an additional 2 h of incubation in gentamicin-containing medium (i.e., at 4 h post-infection), cells and culture supernatants were collected for subsequent analyses. For the time-course experiment, THP-1 cells were infected with *M. bovis* at a fixed MOI of 10. The time of bacterial addition was again defined as 0 hpi. Following the same 2 h of co-incubation, washing and gentamicin treatment procedure as described above, cells were further cultured for 1, 2, or 4 h in gentamicin-containing medium (resulting in total incubation times of 3, 4, and 6 hpi, respectively). Thus, cell and supernatant samples were collected at the indicated time points. Cells cultured with an equal volume of medium served as negative controls. For positive controls, cells were treated with lipopolysaccharide (LPS; 500 ng/mL; Sigma-Aldrich, Germany) for 4 h, followed by washing and stimulation with adenosine triphosphate (ATP; 5 mM, 30 min; Sigma-Aldrich, Germany) to induce NLRP3 inflammasome-mediated pyroptosis. This LPS priming and ATP stimulation protocol was performed in parallel for each assay.

### 2.3. Whole-Transcriptome Sequencing Analysis

#### 2.3.1. Sample Preparation

THP-1 suspension cells were seeded into T75 cell culture flasks (Thermo Fisher, Waltham, MA, USA) at a density of 1 × 10^6^ cells/mL to induce cell adhesion. The culture medium was supplemented to a volume of 10 mL per flask. After 24 h, the medium was replaced with fresh complete medium, and the cells were cultured for an additional 24 h. The experimental design included an uninfected control group (NC_1, NC_2, NC_3) and an *M. bovis*-infected group (Mb_1, Mb_2, Mb_3), each with three biological replicates. THP-1 macrophages were infected with *M. bovis* at an MOI of 10. For sequencing samples, after 2 h of infection, the cell culture medium was replaced with complete medium containing gentamicin, and the cells were cultured for an additional 2 h (i.e., harvested at 4 h post-infection, as defined in the infection protocol). Total cellular RNA was extracted using Trizol reagent (EasyPure^®^ RNA Kit, TransGen Biotech, Beijing, China) and sent to Shanghai Personal Biotechnology Co., Ltd (Personal, Shanghai, China). for whole-transcriptome sequencing analysis.

#### 2.3.2. RNA Quantification and Qualification, Library Construction, and Sequencing

RNA quality and integrity were assessed using agarose gel electrophoresis, NanoDrop 2000 (Thermo Fisher Scientific, Waltham, MA, USA), and an Agilent 2100 Bioanalyzer (Agilent Technologies, Santa Clara, CA, USA). Total RNA with RIN ≥ 8.0 was used for library preparation. Strand-specific mRNA, lncRNA, and circRNA libraries were constructed using the dUTP-based strand-specific library preparation after rRNA depletion. miRNA libraries were prepared using the TruSeq Small RNA Sample Prep Kit (Illumina). Paired-end sequencing (150 bp) was performed on an Illumina HiSeq platform. Clean reads were aligned to the human genome (GRCh38, Ensembl v108) using HISAT2 (v2.1.0). For mRNA, read counts per gene were quantified using HTSeq (v0.9.1) and normalized to fragments per kilobase of transcript per million mapped reads (FPKM). For lncRNA, read counts were quantified at the transcript level using Stringtie (v2.2.1) and normalized to FPKM. For circRNA, read counts were extracted from the find_circ (v1.0) results and normalized to transcripts per million (TPM). For miRNA, read counts were derived from reads aligned to mature miRNA sequences. Differential expression analysis was performed using DESeq2 (v1.38.3 for mRNA/lncRNA/circRNA, and v1.39.0 for miRNA), with |log_2_ fold change| > 1 and FDR < 0.05 as thresholds. All samples were processed in a single batch; no batch correction was required.

#### 2.3.3. ceRNA Network Prediction and Construction

The miRanda software (v3.3a, Memorial Sloan Kettering Cancer Center, Manhattan, New York, NY, USA) was employed to predict target gene (mRNA, lncRNA) pairs associated with miRNAs. The prediction thresholds were set as follows: miRanda score ≥ 140 and minimum free energy (ΔG) ≤ −20 kcal/mol. Expression correlations between lncRNA and mRNA were computed using the Pearson correlation coefficient, with a cutoff of |r| ≥ 0.7 and *p* < 0.05. Subsequently, a hypergeometric test was performed on miRNAs shared by lncRNA and mRNA to assess the lncRNA’s potential as a ceRNA; a *p*-value < 0.05 was considered statistically significant. Regulatory similarity and sensitive correlation scores were derived from the correlations among lncRNAs, miRNAs, and mRNAs, and only those with a sensitive correlation score > 0.3 were retained for further analysis. The predicted miRNA–mRNA and lncRNA–miRNA pairs that simultaneously passed all the above thresholds were compiled into a network and visualized using Cytoscape (v3.9.1, Institute of Systems Biology, Seattle, WA, USA). Final network nodes (lncRNAs, miRNAs, mRNAs) were selected based on the following criteria: (i) the lncRNA and mRNA were significantly correlated (|r| ≥ 0.7, *p* < 0.05); (ii) both the lncRNA–miRNA and miRNA–mRNA interactions were predicted using miRanda with the defined thresholds; and (iii) the hypergeometric test for shared miRNAs was significant (*p* < 0.05).

### 2.4. Reverse Transcription-Quantitative PCR

Total RNA was extracted from THP-1 cells using TRIzol reagent (EasyPure^®^ RNA Kit, TransGen Biotech, Beijing, China), and the concentration and purity were determined using a microplate reader (BioTek, Windsor, VT, USA). RNA integrity was assessed via agarose gel electrophoresis (clear 28S and 18S bands) and confirmed by an RNA integrity number (RIN) ≥ 8.0 obtained using a Bioanalyzer. Complementary DNA (cDNA) for mRNA, lncRNA, and circRNA was prepared using PrimeScript™ RT reagent Kit with gDNA Eraser (Takara, Osaka Prefecture, Osaka City, Japan). Reverse-transcription controls (no-RT controls) were included to rule out genomic DNA contamination. The expression levels were quantified via qPCR using TB Green^®^ Premix Ex TaqTM II (Takara, Japan), with GAPDH as the internal reference. Primer efficiency was validated (90–110%) using a standard curve. Melt curve analysis confirmed a single product for each amplicon, demonstrating amplicon specificity. No-template controls (NTC) were included in each run. The miRNA cDNA was synthesized using the StarScript III miRNA RT Kit (Genstar, Shanghai, China), and its expression was analyzed via qPCR using 2 × RealStar Fast SYBR qPCR Mix (Genstar, China) and U6 as the internal reference. Triplicate samples were analyzed, and relative expression was calculated using the 2^−ΔΔCt^ method. Primers were designed and synthesized by Sangon Biotech (Shanghai, China) Co., Ltd. (mRNA, lncRNA, circRNA) and Vazyme Biotech Co., Ltd. (Nanjing, China) (miRNA), as detailed in Table S1.

### 2.5. Enzyme-Linked Immunosorbent Assay

The levels of IL-1β (BYabscience, Nanjing, China), IL-18 (BYabscience, Nanjing, China), and the protein concentration of lactate dehydrogenase (LDH; BYabscience, BY-EH114560, Nanjing, China) in the cell supernatants were quantified using commercial ELISA kits according to the manufacturer’s protocols. Absorbance was measured at 450 nm using a microplate reader (BioTek, USA).

### 2.6. Caspase-1 Enzyme Activity Assay

Treated cells were collected, and total cell protein was extracted and quantified. Following the Caspase-1 Activity Detection Kit (Beyotime, Shanghai, China) protocol, samples were added to the detection buffer and mixed with 10 μL of Ac-YVAD-pNA (2 mM). The mixture was incubated at 37 °C for 60 to 120 min. Upon noticeable color change, absorbance at 405 nm (A405) was measured using a microplate reader (BioTek, USA). The A405 of the blank control was subtracted from the sample’s A405 to determine the absorbance of pNA produced by caspase-1. The amount of pNA was calculated using a standard curve. According to Chemicon, one unit of caspase-1 activity cleaves 1.0 nmol of Ac-YVAD-pNA per hour at 37 °C under saturated substrate conditions. In this way, the caspase-1 enzyme activity units in the samples could be calculated.

### 2.7. Immunofluorescence Staining

THP-1 cells were seeded into a 48-well culture plate (Thermo Fisher, USA) at a density of 1 × 10^6^ cells/mL to induce adherence. Coverslips were placed at the bottom of the wells in advance. The cells were fixed with 4% paraformaldehyde (Solarbio, China) for 30 min at room temperature and then washed three times with sterile PBS and blotted dry. Cells were permeabilized with 0.1% Triton X-100 (Solarbio, China) for 30 min at room temperature and then blocked with 5% powdered non-fat milk (BD Difco, Franklin Lakes, NJ, USA) for 1 h at 37 °C. Cells were incubated with primary antibody against GSDMD (full length + N terminal, rabbit polyclonal antibody; 1:100; catalog number: A20197; ABclonal, Wuhan, China) overnight at 4 °C. After thorough rinsing, cells were incubated with the secondary antibody (ABclonal, Wuhan, China) for 1 h at 37 °C in the dark. Finally, the cells were stained with 4′,6-diamidino-2-phenylindole (DAPI; Beyotime, Shanghai, China) for 1 min at room temperature in the dark and observed using an inverted fluorescence microscope (Leica, Wetzlar, Germany).

### 2.8. Flow Cytometry Assay

The THP-1 macrophages were resuspended in 295 μL of 1× binding buffer, according to the experimental design and grouping. The cells were incubated at 37 °C for 30 min in the dark with 5 μL of a 1:60 dilution of FLICA^®^ 660 Caspase-1 working solution (ImmunoChemistry, Davis, CA USA) and 5 μL of propidium iodide (PI; Invitrogen, 00-6990-50, Carlsbad, CA, USA). For flow cytometry (BD FACSVerse^TM^, Franklin Lakes, NJ, USA), debris and aggregates were excluded through FSC/SSC and FSC-A/FSC-H gating; at least 10,000 events per sample were collected. Fluorescence compensation was performed using single-stained controls, and gate boundaries were set based on unstained and positive controls. Caspase-1/PI double-positive cells were defined as the pyroptotic cell population. Flow cytometry determined the proportion of pyroptotic cells to quantify the number of caspase-1 and PI-stained cells.

### 2.9. Electron Microscopy Assays

Scanning electron microscopy: Based on the experimental design and grouping, cells affixed to coverslips underwent fixation, dehydration, drying, and vacuum-spraying before being examined with a biological scanning electron microscope (Hitachi Regulus 810, Tokyo, Japan).

Transmission electron microscopy: Cell clusters were collected based on the experimental design and grouping. Following fixation, washing, gradient dehydration, infiltration, embedding, sectioning, staining, and thorough drying, samples were examined using a biological transmission electron microscope (JEOL JEM-2100plus, Toshima City, Tokyo, Japan).

### 2.10. Plasmid Construction

The P2RX7 and MALAT1 sequences were derived from whole-transcriptome sequencing data. Primers for MALAT1 and P2RX7 were designed using an online tool (https://www.takarabio.com/learning-centers/cloning/in-fusion-cloning-tools (accessed on 27 May 2026)). Restriction enzyme recognition sites for *Sma* I (TransGen Biotech, Beijing, China) and *Xho*I (TransGen Biotech, Beijing, China) were incorporated into the PCR primers. The specific primer sequences are provided in Table S2.

Using the remaining RNA samples from sequencing, P2RX7 cDNA was synthesized with StarScript III RT MasterMix (Genstar, China), while MALAT1 cDNA was synthesized using StarScript Pro All-in-one RT Mix with gDNA Remover (Genstar, Beijing, China). These cDNAs served as templates for PCR amplification of the target genes P2RX7 and MALAT1. Following sequencing and purification, the PCR products were ligated into the vectors pEGFP-N2 and pEGFP-C1 via homologous recombination. The recombinant plasmids, pEGFP-N2-P2RX7 and pEGFP-C1-MALAT1, were extracted using the Endotoxin-Free Plasmid Extraction Kit (Omega, Norcross, GA, USA).

### 2.11. Cell Transfection

The pEGFP-C1-MALAT1 and pEGFP-N2-P2RX7 constructs were generated in previous experiments. miR-20b-5p mimic, miR-20b-5p inhibitor, si-MALAT1 (si-MALAT1-1#, si-MALAT1-2#, si-MALAT1-3#), si-P2RX7 (si-P2RX7-1#, si-P2RX7-2#, si-P2RX7-3#, si-P2RX7-4#), negative control si nc, and positive control si nc FAM were designed and synthesized by Shanghai Gemma Gene Co., Ltd (Gemma, Shanghai, China). The specific sequences are provided in Table S3. THP-1 cells were seeded into 6-well plates at a density of 1 × 10^6^ cells per well and treated with PMA to induce adherence; after 24 h, the medium was replaced to remove non-adherent cells, at which point the adherent cells reached 60–80% confluence. The cells were then cultured for an additional 24 h to stabilize their condition. For transfection, nucleic acids were mixed with Advanced DNA RNA Transfection Reagent (AD600025, Zeta Life, San Francisco, CA, USA) at a 1:1 ratio; specifically, 6 µg of plasmid DNA was mixed with 6 µL of the reagent, and 6 µL of siRNA was mixed with 6 µL of the reagent. The mixture was gently pipetted up and down 10–15 times and incubated at room temperature for 15 min to form transfection complexes, following which 12 µL of the mixture was added directly to the cells in each well. After 24 h of incubation, the medium was replaced with 2 mL of fresh complete medium containing 10% fetal bovine serum (without antibiotics). For plasmid DNA transfection, fluorescence was observed under a microscope 48 h after medium replacement; for siRNA transfection, fluorescence was examined 9 h after transfection. Transfection was considered successful when >80% of the cells displayed green fluorescence. Final transfection efficiency was assessed via qPCR, and subsequent infection experiments were performed 48 h after transfection.

### 2.12. Dual-Luciferase Reporter Assay

StarBase (https://rnasysu.com/encori/ (accessed on 27 May 2026)) was employed to predict binding sites between miR-20b-5p and MALAT1, while TargetScan (https://www.targetscan.org/ (accessed on 27 May 2026)) was used for miR-20b-5p and P2RX7. Partial sequences of MALAT1 and P2RX7 with wild-type (WT) and mutant (MUT) miR-20b-5p binding sites were cloned into the pmirGLO luciferase reporter vector, labeled as MALAT1-WT, MALAT1-MUT, P2RX7-WT, and P2RX7-MUT (the wild-type and mutant sequences are provided in Table S4). These constructs (1 μg each), along with miR-20b-5p mimic (40 pmol) or miR-20b-5p NC (40 pmol), were transfected into HEK-293T cells seeded in 12-well plates using 1.6 μL Lipo8000 (Beyotime, China). After 48 h, luciferase activity was measured according to the instructions of the Dual-Luciferase Assay Kit (Promega, Madison, WI, USA), with firefly luciferase activity normalized to Renilla luciferase activity (Firefly/Renilla ratio). All experiments were performed in three technical replicates.

### 2.13. Cell Counting Kit-8 Assay

THP-1 cells were seeded in 96-well plates at a density of 1 × 10^4^ cells/mL to promote adhesion. Following treatment under the different experimental conditions, cells were incubated with 10 µL of CCK-8 reagent (Biosharp, Hefei, China) at 37 °C for 1 h. Absorbance at 450 nm was measured using a microplate reader (BioTek, USA).

### 2.14. Western Blotting Assay

Cells were lysed using pre-cooled RIPA buffer (BestBio, China) with protease and phosphatase inhibitors, and proteins were extracted via centrifugation at 12,000× *g* for 15 min at 4 °C. Protein concentrations were measured using a BCA assay kit (Beyotime, China). Then, 25 μg protein samples were resolved on a 10% SDS-PAGE gel and transferred to methanol-activated PVDF membranes (Millipore Sigma, Darmstadt, Germany). Each membrane was blocked with 5% non-fat milk (BD Difco, USA) for 2 h at room temperature. After washing with 1× PBST, the membranes were incubated overnight at 4 °C with primary antibodies against NLRP3 (1:500; ABclonal, China), Caspase-1 (1:1000; ABclonal, China), GSDMD (1:1000; ABclonal, China), and GAPDH (1:5000; Proteintech, Wuhan, China). Following another wash with 1× PBST, the membranes were incubated with an HRP-conjugated secondary antibody (ABclonal, China) for 1.5 h at room temperature. ECL luminescence reagent (Tanon, Shanghai, China) was added to the PVDF membranes. Then, detection was conducted using a fully automated chemiluminescence image analysis system (Sensi Saichi, Beijing, China).

### 2.15. Statistical Analysis

All experiments were performed once using a single batch of cells, with three technical replicates per group. In this study, “n” refers to the number of technical replicates, and statistical analyses were based on these technical replicates. Data are presented as the mean ± SD of technical replicates. Choice of statistical tests: For comparisons involving only two groups (e.g., NC vs. *M. bovis* infection), an unpaired two-tailed Student’s *t*-test was used. For comparisons involving three or more groups with a single independent variable (e.g., NC vs. PC, MOI 1, MOI 10, MOI 100), a one-way analysis of variance (ANOVA) was performed. When the ANOVA indicated a significant overall effect, Dunnett’s post hoc test was applied to compare each treatment group specifically against the control group. For experiments involving two independent variables (e.g., *M. bovis* infection and time post-infection), a two-way ANOVA was used, followed by Sidak’s multiple comparisons test where appropriate. All statistical tests were two-sided, and a *p*-value < 0.05 was considered statistically significant. Significance levels are indicated as: * *p* < 0.05, ** *p* < 0.01, *** *p* < 0.001. “ns” denotes non-significant differences. Analyses were performed using Prism 8.0 (GraphPad Software Inc., San Diego, CA, USA).

## 3. Results

### 3.1. Establishment of a Pyroptosis Model Induced by M. bovis in THP-1 Cells

**Figure 1:**
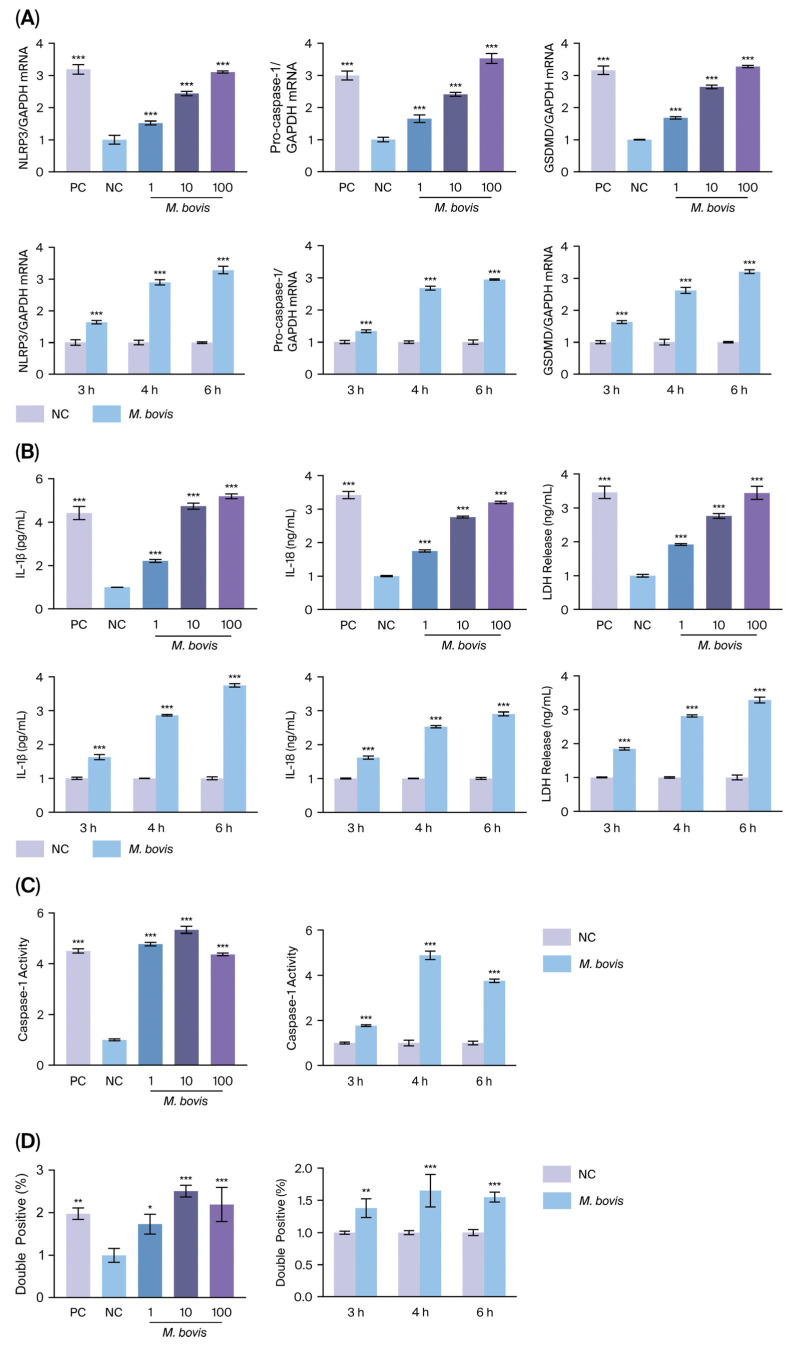

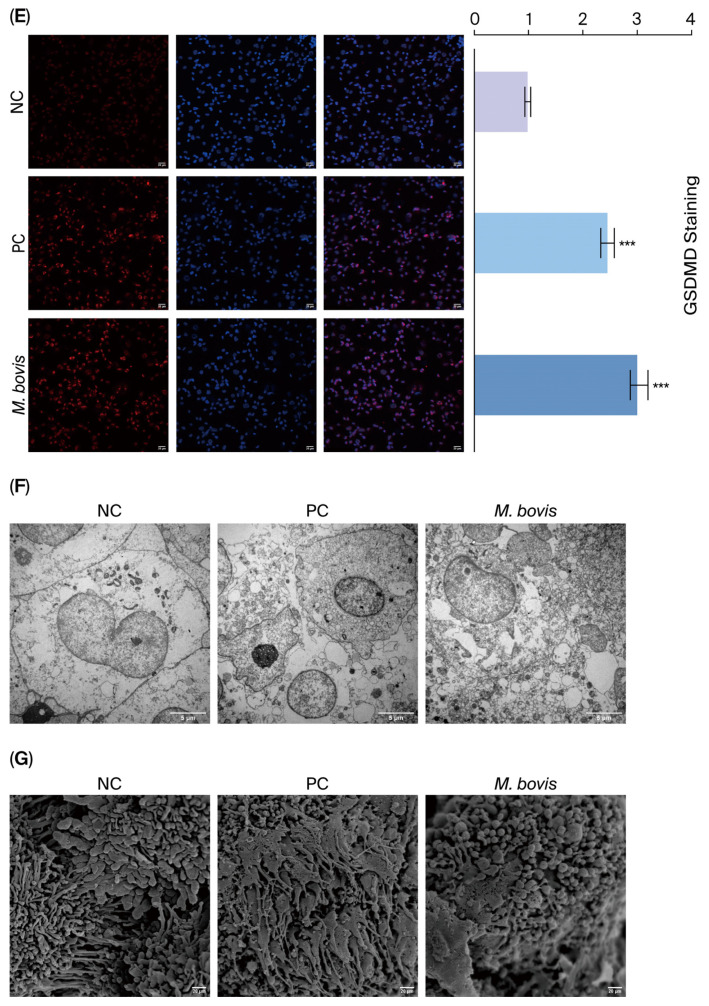
This study established a pyroptosis model in THP-1 cells infected with *M. bovis*. (**A**) demonstrates the qPCR-detected expression levels of pyroptosis-related genes *NLRP3*, *pro-caspase-1*, and *GSDMD* at varying multiplicities of infection (MOI = 1, 10 and 100 for 4 h) and infection durations (MOI = 10 for 3, 4, or 6 h). (**B**) shows ELISA-measured concentrations of pyroptosis-associated cytokines IL-1β and IL-18 and the cell death marker LDH in the cell supernatant at different MOIs and infection times. (**C**) depicts the effects of *M. bovis* on caspase-1 enzyme activity under varying MOIs and infection durations. (**D**) presents the results of the flow cytometry analysis, revealing the proportion of pyroptotic cells (caspase-1 and PI double-positive) upon *M. bovis* infection under different experimental conditions. (**E**) displays dual fluorescent labeling of GSDMD (red) and DAPI (blue) in THP-1 cells under various treatment conditions, showing the GSDMD staining pattern (scale bar = 20 μm). (**F**,**G**) illustrate pyroptosis-related morphological changes in THP-1 cells infected with *M. bovis*, observed via transmission electron microscopy (scale bar = 5 μm) and scanning electron microscopy (scale bar = 20 μm), respectively. Data are presented as the mean ± SD of three technical replicates (n = 3 per group) from a single representative experiment. One-way ANOVA with Dunnett’s post hoc test was used for comparisons of each treatment group against the NC group. For time-course experiments, two-way ANOVA with Sidak’s multiple comparisons test was used (*M. bovis* vs. NC at each time point). Significance levels: * *p* < 0.05, ** *p* < 0.01, *** *p* < 0.001; ns, not significant. MOI refers to the average number of infectious agents per target cell. Accordingly, MOI values of 1, 10, and 100 represent an average of 1, 10, and 100 *M. bovis* per THP-1 cell, respectively. NC indicates the negative control, where cells were untreated, whereas PC indicates the positive control, with cells treated with LPS and ATP.

The assembly of inflammasomes and pore formation in the cell membrane caused by the N-terminal of the execution protein GSDMD are central to pyroptosis. qPCR analysis revealed that the expression levels of the pyroptosis-related genes *NLRP3*, *pro-caspase-1*, and *GSDMD* were significantly upregulated in the infected and positive control groups compared with the negative controls. Furthermore, the upregulation of these genes increased with the MOI and infection duration (Figure 1A).

IL-1β and IL-18 are downstream markers of pyroptosis that are released extracellularly through membrane pores, which initiate a local inflammatory response and signal the recruitment of immune cells for immunomodulation. LDH indicates cell membrane integrity, as its extracellular release during pyroptosis reflects the severity of cell damage. ELISA analysis demonstrated that IL-1β, IL-18, and LDH concentrations were significantly elevated in the infected and positive control groups compared with the negative controls, with levels increasing in a manner dependent on infection multiplicity and duration (Figure 1B).

Inflammasome assembly promotes cleavage of pro-caspase-1 into active caspase-1. The infected and positive control groups exhibited significantly elevated caspase-1 activity compared with the negative control group. Among the varying MOI conditions, the group with an MOI of 10 exhibited the highest caspase-1 activity. Additionally, the infection group at 4 h post-infection showed the greatest increase in caspase-1 activity (Figure 1C).

Flow cytometric analysis of THP-1 cells exposed to *M. bovis* revealed an increased proportion of cells undergoing pyroptosis, as indicated by elevated caspase-1/PI double-positive staining, in the infected and positive control groups compared with the negative controls. The highest pyroptosis rate was observed at an MOI of 10. Furthermore, at 4 h post-infection, the infected group exhibited the highest degree of pyroptosis (Figure 1D; original flow cytometry plots are shown in Figure S4).

Infection at an MOI of 10 for 4 h significantly altered the expression of pyroptosis-related genes. Cellular immunofluorescence analysis revealed that GSDMD staining was substantially elevated in the infected and positive control groups compared with the negative controls (Figure 1E).

Transmission electron microscopy analysis revealed ultrastructural alterations in infected cells, including disruption of nuclear and plasma membranes, organelle injury, and leakage of cellular contents (Figure 1F). Scanning electron microscopy further demonstrated that the infected cells exhibited swelling, formation of small vesicles and pores on the villi, and compromised cell membrane integrity (Figure 1G). Based on these results, this study employed a pyroptosis model in which THP-1 cells were infected with *M. bovis* at an MOI of 10 for 4 h.

### 3.2. Whole-Transcriptome Sequencing Analysis of THP-1 Cells Infected by M. bovis

We conducted whole-transcriptome sequencing on infected samples to investigate RNA expression changes regulating *M. bovis*-induced pyroptosis in THP-1 cells. As illustrated in Figure S1A–D, we identified 741 differentially expressed mRNAs, with 465 upregulated and 276 downregulated. Additionally, 1049 differentially expressed lncRNAs were observed, with 531 upregulated and 518 downregulated. The analysis also revealed 25 differentially expressed circRNAs, with 15 upregulated and 10 downregulated; furthermore, 40 differentially expressed miRNAs were found, including 3 upregulated and 37 downregulated. Several differentially expressed mRNAs, lncRNAs, circRNAs, and miRNAs are described in Table S5.

The Gene Ontology (GO) enrichment analysis of differentially expressed genes is visualized using bar and bubble charts (Figure S2A–D). Differentially expressed mRNAs were primarily associated with functions related to cell migration, inflammatory responses, immune system processes, and the regulation of multicellular organismal processes. Target genes of differentially expressed lncRNAs were enriched in key immunity-related pathways, including immune response-regulating signaling, antigen receptor-mediated signaling, immune response-activating cell surface receptor signaling, immune response-activating signal transduction, and immune response activation. Source genes for differentially expressed circRNAs were abundant in functions related to postsynaptic membranes, regulation of plasma membrane-bound cell projection organization, dendritic spines, and regulation of cell projection organization. The target genes of differentially expressed miRNAs were predominantly enriched in functions involving ion and cation binding, metal ion binding, and cellular metabolic processes.

A Kyoto Encyclopedia of Genes and Genomes (KEGG) enrichment analysis of differentially expressed genes was conducted, with the results depicted as bar and bubble charts (Figure S3A–D). The analysis revealed that differentially expressed mRNAs were primarily enriched in the TNF, NF-κB, Toll-like receptor, and NOD-like receptor signaling pathways. The target genes of differentially expressed lncRNAs showed enrichment in NF-κB, Toll-like receptor, and NOD-like receptor pathways. Genes associated with differentially expressed circRNAs were enriched in pathways such as Fc gamma R-mediated phagocytosis, NF-κB, and protein processing in the endoplasmic reticulum. Furthermore, the target genes of differentially expressed miRNAs were enriched in critical signaling pathways, including TGF-beta, calcium, and Rap1, which are essential for cell immune regulation and implicated in the pathogenesis of various diseases.

**Figure 2:**
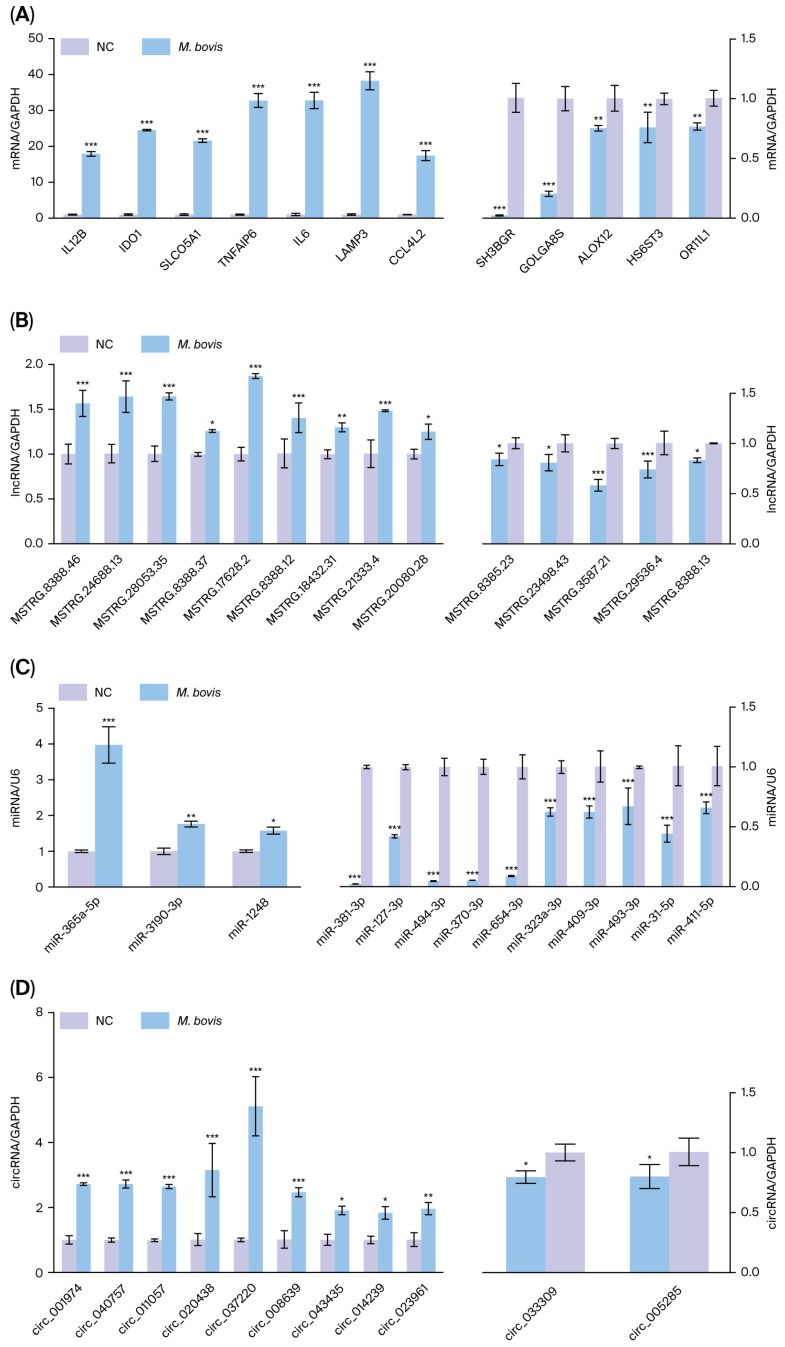
qPCR detection results for differentially expressed RNAs. (**A**–**D**) display the qPCR outcomes for mRNA, lncRNA, miRNA, and circRNA, respectively. Significance levels are indicated as: * *p* < 0.05, ** *p* < 0.01, *** *p* < 0.001.

Twelve differentially expressed mRNAs, 14 lncRNAs, 13 miRNAs, and 11 circRNAs were chosen from the sequencing data for qPCR analysis. The qPCR results (Figure 2A–D) aligned with the omics sequencing data, confirming the stability and reliability of the sequencing results.

### 3.3. Construction and Analysis of the lncRNA-miRNA-mRNA Regulatory Network

**Figure 3:**
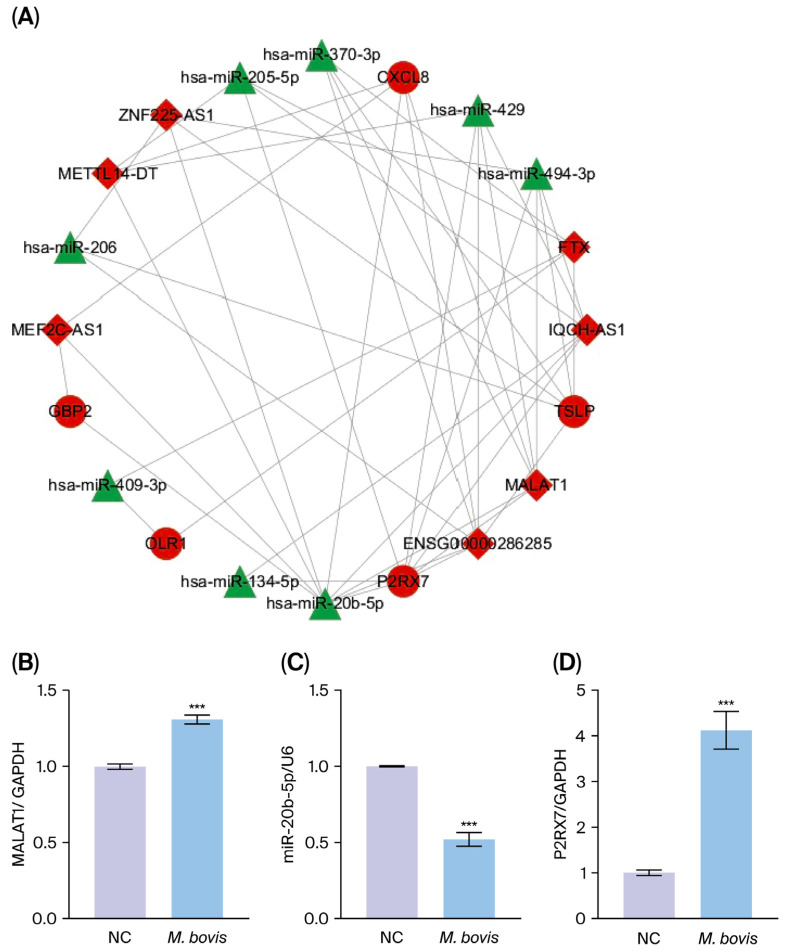
The lncRNA–miRNA–mRNA regulatory network was established post-*M. bovis*-infection of THP-1 cells, and the expression levels of *MALAT1*, miR-20b-5p, and *P2RX7* were confirmed. (**A**) depicts the putative lncRNA–miRNA–mRNA network, where circles represent differentially expressed mRNAs, triangles represent differentially expressed miRNAs, and diamonds represent differentially expressed lncRNAs. Upregulated genes are shown in red, and downregulated genes are shown in green. (**B**–**D**) present the qPCR validation of *MALAT1*, miR-20b-5p, and *P2RX7* expression following *M. bovis* infection of THP-1 cells. Significance levels are indicated as: *** *p* < 0.001.

Whole-transcriptome sequencing and bioinformatics analysis identified a potential lncRNA–miRNA–mRNA regulatory network comprising 187 differentially expressed genes: 118 lncRNAs (99 upregulated and 19 downregulated), 22 miRNAs (2 upregulated and 20 downregulated), and 47 mRNAs (41 upregulated and 6 downregulated) (Tables S6–S8). Based on these overlapping differentially expressed lncRNAs, miRNAs, and mRNAs, a putative ceRNA regulatory network was constructed, with only representative genes displayed (Figure 3A). The activation of NOD-like receptors is closely linked to inflammasome formation and pyroptosis, playing a crucial role in the body’s immune defense system. Investigating the interactions within this network provided deeper insights into immune regulation and the inflammatory process. Consequently, the potential ceRNA axis, MALAT1–miR-20b-5p–P2RX7, was selected for additional mechanistic investigation.

The qPCR analysis revealed that, in *M. bovis*-infected THP-1 macrophages, *MALAT1* expression was significantly upregulated (Figure 3B), miR-20b-5p expression was significantly downregulated (Figure 3C), and *P2RX7* expression was significantly upregulated (Figure 3D) when compared with the uninfected group. These findings align with the whole-transcriptome sequencing data, suggesting a potential association of MALAT1, P2RX7, and miR-20b-5p with *M. bovis*-induced pyroptosis in THP-1 cells.

### 3.4. MALAT1 Downregulation Alleviated M. bovis-Mediated THP-1 Cells Pyroptosis

**Figure 4:**
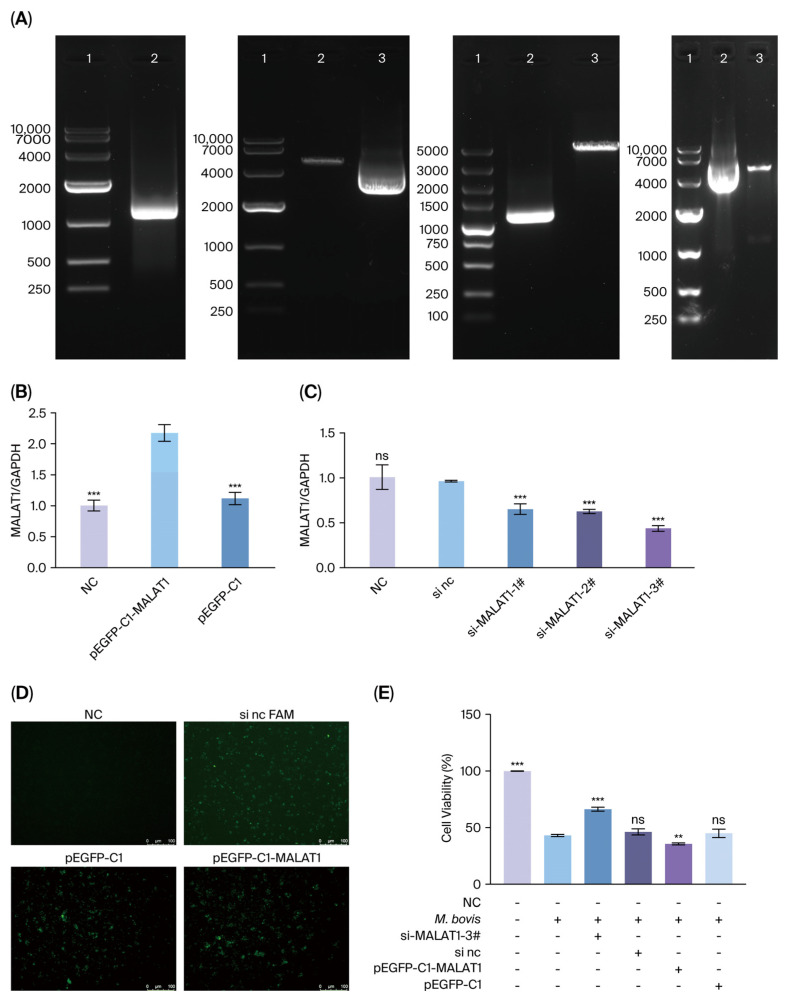

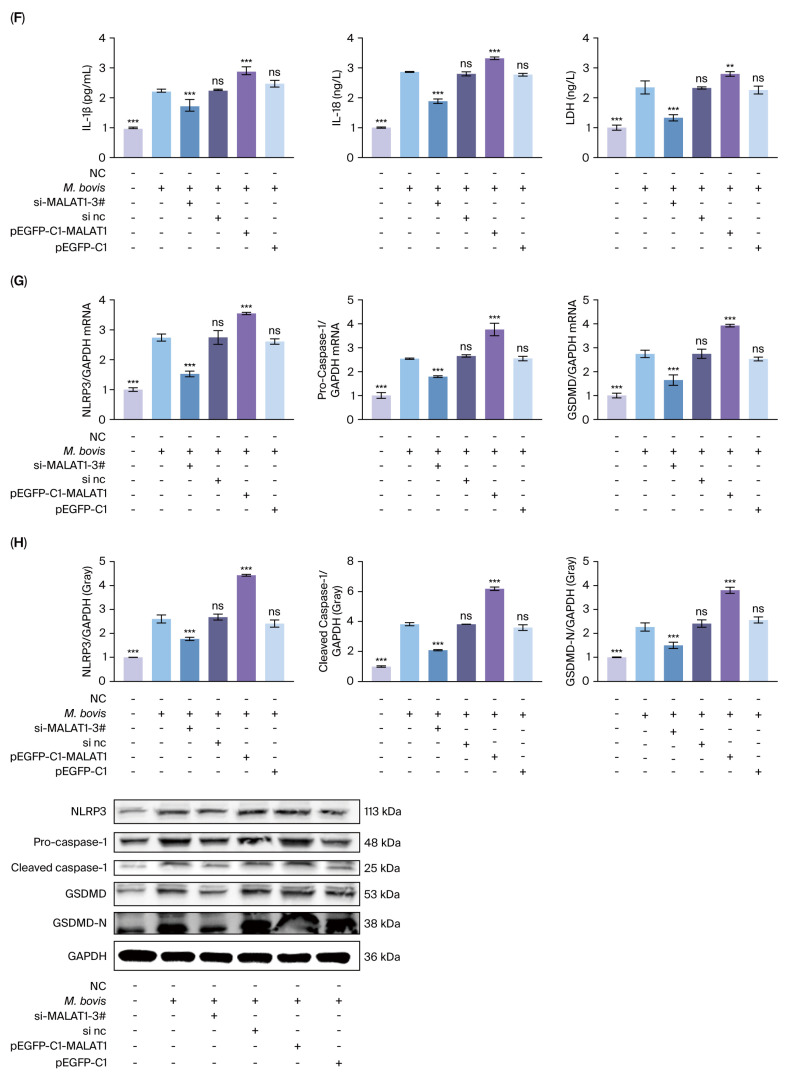
MALAT1 regulates *M. bovis*-induced pyroptosis in THP-1 cells. To investigate this, MALAT1 overexpression and knockdown vectors were developed. (**A**) illustrates the construction of the recombinant plasmid pEGFP-C1-MALAT1, sequentially showing *MALAT1* gene amplification (1: DL10000, 2: MALAT1); double-enzyme digestion and identification of the pEGFP-C1 vector (1: DL10000, 2: digested pEGFP-C1, 3: pEGFP-C1 plasmid); and identification of purified pEGFP-C1 and MALAT1 products (1: DL5000, 2: MALAT1, 3: pEGFP-C1). It also includes the double-enzyme digestion of the endotoxin-free pEGFP-C1-MALAT1 plasmid (1: DL10000, 2: pEGFP-C1-MALAT1, 3: digested product). (**B**) shows that the transfection efficiency of the pEGFP-C1-MALAT1 overexpression plasmid was confirmed via qPCR. (**C**) shows that the knockdown efficacy of three MALAT1 interference sequences was validated via qPCR, with si nc as the negative control. (**D**) reveals that fluorescence in THP-1 cells transfected with the overexpression vector and interference sequence was observed, with si nc FAM confirming successful interference sequence transfection. (**E**) indicates the effect of MALAT1 on the viability of THP-1 cells infected with *M. bovis*. (**F**) shows the impact of MALAT1 on IL-1β and IL-18 secretion and the release of LDH in the supernatant of THP-1 cells infected with *M. bovis*. Furthermore, (**G**,**H**) indicate the effect of MALAT1 on the expression level of pyroptosis-related genes and proteins in *M. bovis*-infected THP-1 cells, respectively. Significance levels are indicated as: ** *p* < 0.01, *** *p* < 0.001. “ns” denotes non-significant differences.

The impact of MALAT1 overexpression and knockdown on *M. bovis*-induced pyroptosis in THP-1 cells was investigated. Gel electrophoresis confirmed the successful construction of the overexpression plasmid pEGFP-C1-MALAT1 (Figure 4A; original gel electrophoresis images see Figure S5). Transfection of this recombinant plasmid and the empty vector confirmed transfection efficiency. The qPCR analysis revealed a significant increase in *MALAT1* expression in the pEGFP-C1-MALAT1-transfected group compared with the negative control and pEGFP-C1-transfected groups (Figure 4B). Three siRNAs that specifically target MALAT1 were designed, and their application resulted in a significant reduction in *MALAT1* expression in the siRNA-transfected groups compared with the negative controls, with si-MALAT1-3# showing the largest decrease (Figure 4C). Immunofluorescence staining further validated the successful transfection of the overexpression plasmid pEGFP-C1-MALAT1 and the specific siRNA into the cells (Figure 4D).

The effects of MALAT1 overexpression and knockdown on *M. bovis*-induced pyroptosis in THP-1 cells were assessed by measuring cell viability, IL-1β, IL-18, and LDH secretion, as well as pyroptosis-related gene and protein expression. Inhibiting MALAT1 expression mitigated the *M. bovis*-induced decrease in cell viability, while overexpression exacerbated it (Figure 4E). Similarly, MALAT1 inhibition reduced the *M. bovis*-induced increase in IL-1β, IL-18, and LDH secretion, whereas overexpression amplified it (Figure 4F). Additionally, MALAT1 knockdown attenuated the upregulation of *NLRP3*, *pro-caspase-1*, and *GSDMD* expression induced by *M. bovis*, whereas overexpression enhanced it (Figure 4G). Furthermore, MALAT1 knockdown significantly suppressed the *M. bovis*-induced upregulation of NLRP3, cleaved caspase-1, and GSDMD-N-terminal protein expression, while overexpression significantly elevated it (Figure 4H; uncropped original western blot images see Figure S6). These findings reveal that reducing MALAT1 expression mitigated *M. bovis*-induced pyroptosis in THP-1 cells, whereas its overexpression intensified the process.

### 3.5. miR-20b-5p Overexpression Alleviated M. bovis-Mediated THP-1 Cells Pyroptosis

**Figure 5:**
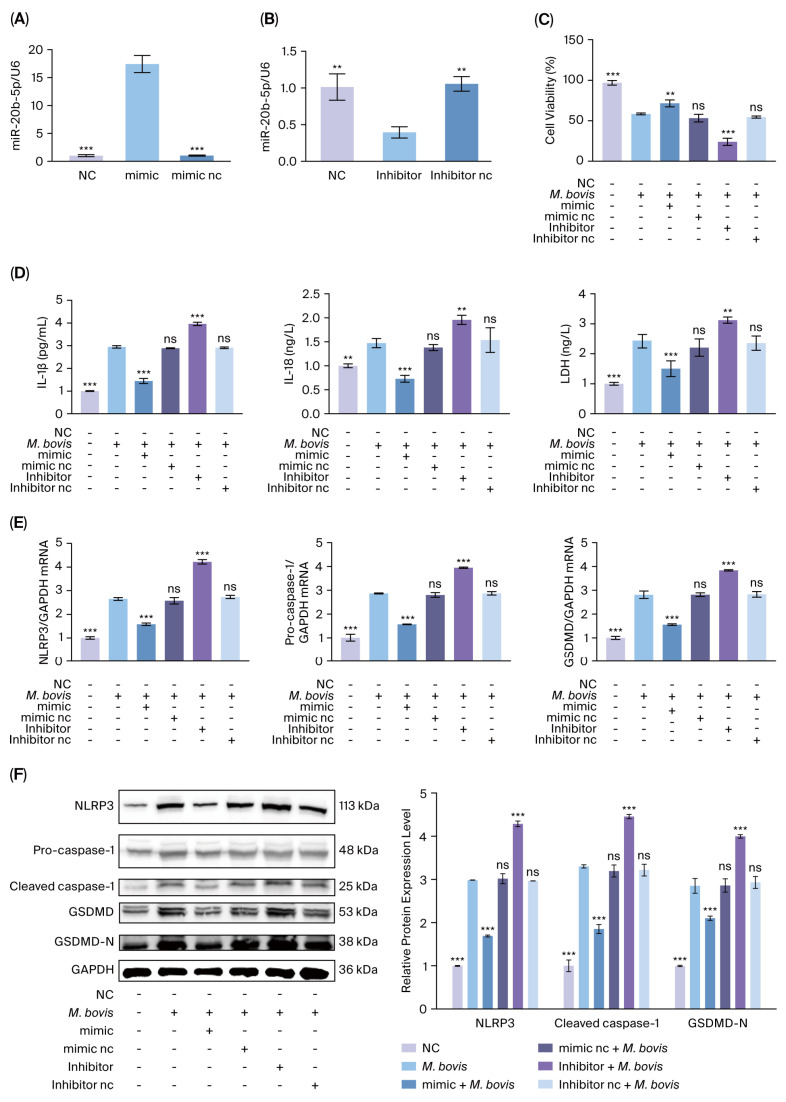
miR-20b-5p modulates *M. bovis*-induced pyroptosis in THP-1 cells. (**A**) shows that qPCR analysis confirmed the overexpression of miR-20b-5p following transfection with the miR-20b-5p mimic. (**B**) indicates that qPCR analysis confirmed the downregulation of miR-20b-5p after transfection with the miR-20b-5p inhibitor in THP-1 cells. (**C**) shows the impact of miR-20b-5p on cell viability in *M. bovis*-infected THP-1 cells. (**D**) indicates the fluorescence validation of the miR-20b-5p mimic and inhibitor (si nc FAM). Furthermore, the effect of miR-20b-5p on the secretion of IL-1β and IL-18, as well as the release of LDH, in the supernatant of *M. bovis*-infected THP-1 cells is shown. (**E**,**F**) reveal the influence of miR-20b-5p on the expression levels of pyroptosis-related genes and proteins in *M. bovis*-infected THP-1 cells, respectively. Significance levels are indicated as: ** *p* < 0.01, *** *p* < 0.001. “ns” denotes non-significant differences.

The role of miR-20b-5p in *M. bovis*-induced pyroptosis of THP-1 cells was also clarified. Cells were transfected with a miR-20b-5p mimic or inhibitor, and transfection efficiency was confirmed via qPCR. Compared with the negative controls, miR-20b-5p expression was significantly increased with the mimic (Figure 5A) and decreased with the inhibitor (Figure 5B). The transfected cells were used for further investigation.

Cell viability assays revealed that the miR-20b-5p mimic mitigated the reduction in cell viability due to *M. bovis* infection. By contrast, the miR-20b-5p inhibitor enhanced the effect (Figure 5C). Similarly, the miR-20b-5p mimic decreased the secretion of IL-1β, IL-18, and LDH, whereas the inhibitor increased the levels post-infection (Figure 5D). Furthermore, the miR-20b-5p mimic attenuated the upregulation of *NLRP3*, *pro-caspase-1*, and *GSDMD* expression, whereas the inhibitor enhanced it (Figure 5E). Additionally, the miR-20b-5p mimic significantly suppressed the elevation of NLRP3, cleaved caspase-1, and GSDMD-N-terminal protein levels, in contrast to the enhancement observed with the inhibitor (Figure 5F; uncropped original western blot images see Figure S6). These results demonstrated that miR-20b-5p overexpression suppressed *M. bovis*-induced pyroptosis in THP-1 cells, whereas miR-20b-5p knockdown enhanced the process.

### 3.6. P2RX7 Downregulation Mitigated M. bovis-Mediated THP-1 Cells Pyroptosis

**Figure 6:**
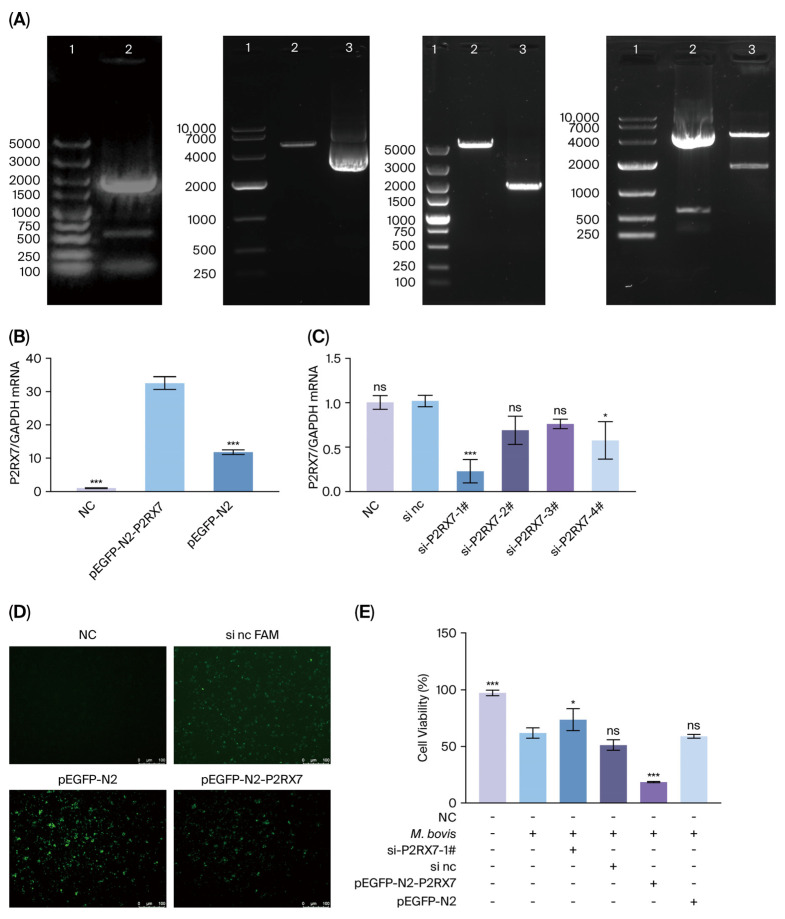

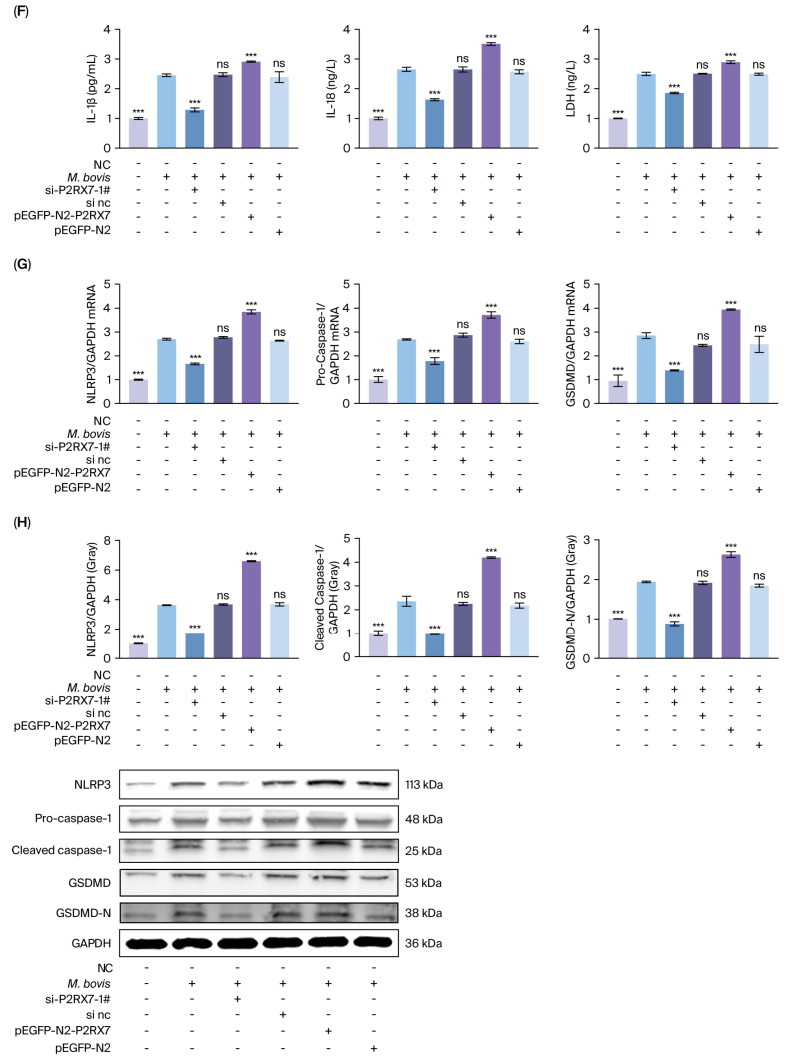
The regulatory role of P2RX7 in *M. bovis*-induced pyroptosis of THP-1 cells was investigated by constructing P2RX7 overexpression and knockdown vectors. (**A**) details the construction of the recombinant plasmid pEGFP-N2-P2RX7. The panel of images from left to right: amplification of the P2RX7 target gene (1: DL5000, 2: P2RX7), double digestion and identification of vector pEGFP-N2 (1: DL10000, 2: double digestion product, 3: pEGFP-N2 plasmid), identification of purified pEGFP-N2 and P2RX7 products (1: DL5000, 2: pEGFP-N2, 3: P2RX7), and double digestion of the endotoxin-free pEGFP-N2-P2RX7 plasmid (1: DL10000, 2: pEGFP-N2-P2RX7 plasmid, 3: double digestion product). (**B**) shows confirmation of overexpression of the *P2RX7* gene via qPCR. (**C**) illustrates the knockdown effects of four P2RX7 interference sequences, validated via qPCR, with si nc as the negative control. (**D**) shows the fluorescence expression of THP-1 cells transfected with the P2RX7 overexpression vector and interference sequences, with si nc FAM as the positive control for interference sequences. (**E**) illustrates the effect of P2RX7 on the viability of THP-1 cells following *M. bovis* infection. (**F**) presents the impact of P2RX7 on the secretion of IL-1β and IL-18, as well as the release of LDH in the supernatant of *M. bovis*-infected THP-1 cells. (**G**,**H**) reveal the effect of P2RX7 on the expression levels of pyroptosis-related genes and proteins, respectively, in THP-1 cells after *M. bovis* infection. Significance levels are indicated as: * *p* < 0.05, *** *p* < 0.001. “ns” denotes non-significant differences.

The role of P2RX7 in *M. bovis*-induced pyroptosis in THP-1 cells was investigated using overexpression and knockdown experiments. Gel electrophoresis confirmed the successful construction of the pEGFP-N2-P2RX7 overexpression plasmid (Figure 6A; original gel electrophoresis images see Figure S5). Transfection of the recombinant plasmid significantly increased *P2RX7* expression compared with the negative control and empty vector groups (Figure 6B). Four siRNAs that specifically target P2RX7 were designed and transfected into THP-1 cells, with si-P2RX7-1# exhibiting the most pronounced downregulation of *P2RX7* expression (Figure 6C). Consequently, si-P2RX7-1# was selected for further study. Immunofluorescence staining confirmed the effective transfection of the pEGFP-N2-P2RX7 plasmid and the specific siRNA into the cells (Figure 6D).

The effects of P2RX7 overexpression and knockdown on *M. bovis*-induced pyroptosis in THP-1 cells were assessed using cell viability assays and measurement of IL-1β, IL-18, and LDH secretion, as well as expression analysis of pyroptosis-related genes and proteins. Silencing P2RX7 alleviated the *M. bovis*-induced decrease in cell viability, whereas its overexpression intensified the reduction (Figure 6E). Furthermore, P2RX7 knockdown significantly lowered IL-1β and IL-18 secretion and LDH release, while overexpression significantly increased these parameters (Figure 6F). Suppressing P2RX7 expression also reduced *NLRP3*, *pro-caspase-1*, and *GSDMD* levels, whereas enhancing P2RX7 expression elevated their levels (Figure 6G). Moreover, P2RX7 knockdown notably decreased the protein levels of NLRP3, cleaved caspase-1, and the N-terminal of GSDMD, while overexpression significantly increased the protein levels (Figure 6H; uncropped original western blot images see Figure S6). These results revealed that P2RX7 knockdown reduced *M. bovis*-induced pyroptosis in THP-1 cells, and P2RX7 overexpression intensified the process.

### 3.7. miR-20b-5p, A Target of MALAT1, Targets P2RX7

**Figure 7:**
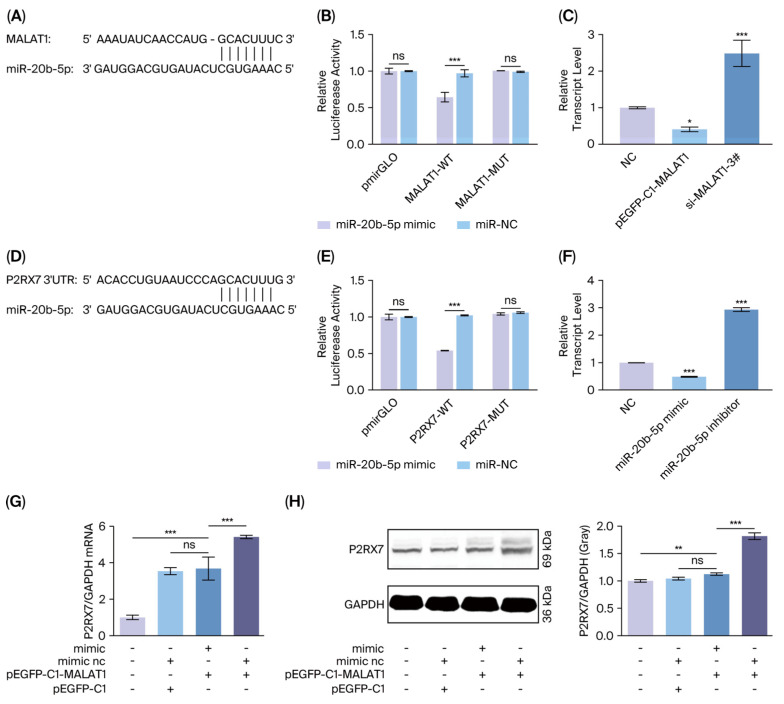
miR-20b-5p targets P2RX7 in THP-1 cells and is regulated by MALAT1. (**A**) identifies the predicted binding sites between MALAT1 and miR-20b-5p. (**B**) demonstrates that dual-luciferase assays confirmed the interaction between MALAT1 and miR-20b-5p. (**C**) indicates that qPCR analysis showed changes in miR-20b-5p expression following MALAT1 overexpression or knockdown. (**D**) shows that binding sites between miR-20b-5p and P2RX7 were identified. (**E**) indicates that dual-luciferase assays validated the interaction between miR-20b-5p and P2RX7. (**F**) shows that qPCR was used to assess *P2RX7* expression in THP-1 cells after transfection with miR-20b-5p mimic or inhibitors. (**G**,**H**) demonstrate that qPCR and Western blotting, respectively, confirmed that miR-20b-5p regulates P2RX7 expression via MALAT1 binding. PmirGLO denotes the empty vector, WT indicates the wild type, and MUT indicates the mutant type. Significance levels are indicated as: * *p* < 0.05, ** *p* < 0.01, *** *p* < 0.001. “ns” denotes non-significant differences.

Bioinformatics analysis of the whole-transcriptome sequencing output identified miR-20b-5p as a potential target of MALAT1. Using starBase (https://rnasysu.com/encori/ (accessed on 27 May 2026)), putative binding sites between miR-20b-5p and MALAT1 were predicted (Figure 7A). The results of a dual-luciferase reporter assay confirmed the interaction, as the miR-20b-5p mimic significantly reduced luciferase activity in the MALAT1-WT reporter but not in the MALAT1-MUT reporter (Figure 7B). Furthermore, qPCR analysis revealed that MALAT1 overexpression significantly decreased miR-20b-5p levels, while MALAT1 silencing increased them (Figure 7C). These findings demonstrate that miR-20b-5p was negatively regulated by MALAT1, confirming it as a target.

The online prediction tool TargetScan (https://www.targetscan.org/ (accessed on 27 May 2026)) was utilized to identify the putative binding site between miR-20b-5p and P2RX7 (Figure 7D). Subsequently, the results of a dual-luciferase reporter assay demonstrated that the miR-20b-5p mimic significantly reduced the fluorescence of the P2RX7-WT reporter, whereas the P2RX7-MUT reporter was unaffected (Figure 7E). The qPCR analysis confirmed that the miR-20b-5p mimic significantly decreased *P2RX7* expression, while the miR-20b-5p inhibitor significantly increased expression (Figure 7F). These results indicate that *P2RX7* is a target gene of miR-20b-5p, which negatively regulates P2RX7 via its binding site.

We employed a miR-20b-5p mimic to elucidate the intermediary role of miR-20b-5p in the MALAT1–miR-20b-5p–P2RX7 ceRNA network. qPCR analysis revealed that co-transfection with the miR-20b-5p mimic nc and the pEGFP-C1-MALAT1 construct significantly upregulated *P2RX7* expression. Conversely, co-transfection with a miR-20b-5p mimic and pEGFP-C1-MALAT1 substantially attenuated this effect (Figure 7G). Furthermore, the Western blot results were consistent with the qPCR results (Figure 7H). These findings indicate that the miR-20b-5p mimic mitigated the upregulation of P2RX7 induced by MALAT1 overexpression, which revealed the intermediary role of miR-20b-5p within the MALAT1–miR-20b-5p–P2RX7 ceRNA axis.

### 3.8. ceRNA Regulatory Mechanism in M. bovis-Mediated THP-1 Cells Pyroptosis

**Figure 8:**
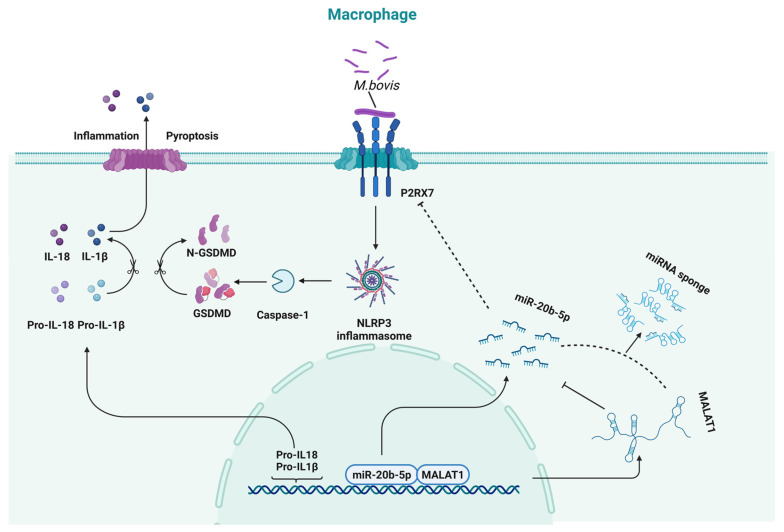
A schematic model demonstrating the role of the MALAT1–miR-20b-5p–P2RX7 axis in pyroptosis of THP-1 cells infected with *M. bovis*.

Our findings demonstrated that MALAT1 functioned as a miRNA “sponge” to modulate miR-20b-5p expression. miR-20b-5p targeted the P2RX7 gene, influencing pyroptosis in THP-1 cells during *M. bovis* infection (Figure 8).

## 4. Discussion

Tuberculosis remains a formidable infectious disease. The zoonotic aspect—particularly *M. bovis* transmission from cattle to humans—poses significant public health challenges, necessitating a deeper understanding of its infection mechanisms. While extensive studies on *M. tuberculosis* have revealed a complex interplay with pyroptosis, the extent to which these mechanisms are conserved in *M. bovis* infection has remained largely unexplored. Based on the high genomic homology (>99.9%) [2] and conserved inflammasome–pyroptosis pathway, it is reasonable to hypothesize that *M. bovis* employs similar strategies to modulate host cell pyroptosis. However, critical differences—such as host tropism [14], virulence gene expression patterns, and epidemiological contexts—warrant direct investigation of *M. bovis*-specific mechanisms.

In *M. tuberculosis* infection, the secreted protein EST12 induces macrophage pyroptosis via RACK1 binding, representing a pathogenic strategy to sustain chronic infection [15]. Conversely, *M. tuberculosis* also employs negative regulators, such as lnc-EST12 and protein phosphatase PtpB, to suppress inflammasome assembly and GSDMD-mediated pyroptosis, facilitating bacterial survival [15,16]. These findings suggest a balanced manipulation of pyroptosis by *M. tuberculosis*. For *M. bovis*, emerging evidence indicates activation of the NLRP3 inflammasome and GSDMD cleavage in infected bovine macrophages and THP-1 cells [11,12]. In the present study, we directly demonstrated that *M. bovis* infection of THP-1 cells activates the NLRP3 inflammasome, producing caspase-1, IL-18, IL-1β, and LDH, with significant changes in pyroptosis-related gene expression as early as 4 h post-infection at an MOI of 10, accompanied by morphological alterations. These results establish a robust in vitro model for studying *M. bovis*-induced pyroptosis and provide the first temporal profile of early pyroptotic events specific to *M. bovis* infection.

THP-1 cells are a well-characterized human monocytic leukemia-derived line and have emerged as the preferred model for investigating pyroptosis in inflammation and immune disorders. These cells exhibit macrophage-like properties and can be readily differentiated into a macrophage-like phenotype using PMA, effectively recapitulating the central role of macrophages in the human immune system. Importantly, THP-1 cells reliably respond to stimuli such as LPS, consistently activating pyroptosis-related signaling pathways [17]. Compared with primary macrophages, THP-1 cells offer advantages in terms of standardized handling, ease of culture, proliferation, and cryopreservation, thereby minimizing experimental variability and making them an ideal model system for mechanistic studies of pyroptosis [17]. However, prior to this study, the mechanism by which *M. bovis* activates pyroptosis in THP-1 cells remained unclear. Our work fills this gap by establishing a defined *M. bovis* infection model and characterizing the early molecular events.

Whole-transcriptome sequencing reveals all transcribed genes and their expression levels, offering a comprehensive view of gene expression in response to pathogens [18]. Frequently observed ncRNAs include miRNAs, lncRNAs, and circRNAs. Previously dismissed as “genetic junk,” advances in omics and numerous studies have shown that these carry out diverse regulatory roles in immune responses and disease progression. The lncRNAs and circRNAs also function as “sponges” for miRNAs via competitive binding, forming a ceRNA network that influences gene expression by regulating target gene stability or translation [19]. Although differential expression of ncRNAs in macrophages during *M. tuberculosis* infection has been reported [20,21], no such comprehensive analysis has previously been performed for *M. bovis*. Here, we performed whole-transcriptome sequencing and bioinformatic analysis of THP-1 macrophages infected with *M. bovis* to elucidate these mechanisms. We identified 741 differentially expressed mRNAs, 1049 differentially expressed lncRNAs, 40 differentially expressed miRNAs, and 25 differentially expressed circRNAs. We then constructed a ceRNA regulatory network composed of 47 mRNAs, 22 miRNAs, and 118 lncRNAs. This represents the first ceRNA network specifically associated with *M. bovis*-induced pyroptosis, providing a valuable resource for understanding host–pathogen interactions unique to zoonotic TB. Additional investigation of key nodes within this network could elucidate novel regulatory mechanisms and identify potential diagnostic or therapeutic targets.

Research has demonstrated that MALAT1 plays a critical role in the pyroptosis of various cell types and regulates multiple diseases through its interactions with miRNAs. For instance, MALAT1 is aberrantly expressed in diabetic nephropathy, with pyroptosis and impaired redox identified as potential pathogenic mechanisms [22]. In the context of diabetic kidney disease, MALAT1 interacts with miR-30c, leading to the upregulation of *NLRP3* and the induction of pyroptosis in HK-2 cells [23]. Furthermore, MALAT1 is overexpressed and miR-141-3p is decreased in high glucose-treated H9C2 cardiomyocytes, suggesting that MALAT1 plays a role in the pathogenesis of diabetic cardiomyopathy. MALAT1 targets miR-141-3p, promoting pyroptosis in these cells [24]. In endotoxemia, MALAT1 is overexpressed and acts as a “sponge” for miR-433-3p, leading to RPTOR upregulation and the inhibition of LPS-activated autophagy in HUVEC cells [25]. This enhances pyroptosis and increases the inflammatory activation of vascular endothelial cells, thereby contributing to the progression of endotoxemia. MALAT1 has also been implicated in the pathogenesis of atherosclerosis. In ox-LDL-treated HUVECs, MALAT1 exerts a protective role by sponging miR-216a-5p and regulating the autophagy-related protein Beclin-1 [26]. Conversely, MALAT1 promotes pyroptosis of EA.hy926 endothelial cells in response to high glucose levels, potentially by competitively binding to miR-22 and affecting the expression of the NLRP3 inflammasome [27]. Furthermore, MALAT1 is involved in TNFα-mediated pyroptosis of RAOECs by regulating the miR-30c-5p/Cx43 axis [28]. These findings highlight the diverse and context-dependent roles of MALAT1 in regulating cellular pyroptosis processes. However, the role of MALAT1 in *M. bovis* infection has not been previously investigated. Our study is the first to demonstrate that MALAT1 is upregulated during *M. bovis*-induced pyroptosis in THP-1 cells. Conversely, silencing MALAT1 mitigated pyroptosis, indicating a pro-pyroptotic role for MALAT1 in this context—a finding that aligns with its function in most inflammatory diseases but contrasts with its protective role in atherosclerosis [26], underscoring the cell type and stimulus-specific nature of MALAT1 activity. Additionally, specific lncRNAs can modulate miRNA expression via a “sponge-like” mechanism. Employing whole-transcriptome sequencing and bioinformatics analyses, miR-20b-5p was identified as the target miRNA of MALAT1. The starBase prediction software identified binding sites between MALAT1 and miR-20b-5p, which were confirmed using dual-luciferase reporter assays and qPCR. Notably, the interaction between MALAT1 and miR-20b-5p has been reported in other diseases, such as chronic hepatitis B [29] and retinoblastoma [30]. Our finding that this axis is also operative in *M. bovis*-infected macrophages suggests a conserved regulatory module that may be broadly exploited in infectious and inflammatory conditions. This cross-disease conservation strengthens the rationale for MALAT1–miR-20b-5p targeting as a therapeutic strategy, potentially extending beyond tuberculosis.

miR-20b-5p plays an essential role in the progression of various diseases, including regulating essential biological processes, such as apoptosis, pyroptosis, proliferation, migration, and polarization, in diverse cell types. In LPS-treated Caco2 cells, miR-20b-5p is a target gene of circDMNT3B, and silencing circDMNT3B enhances cell viability, reduces apoptosis, and alleviates intestinal mucosal permeability [31]. However, these effects are significantly inhibited by miR-20b-5p overexpression. In retinoblastoma, miR-20b-5p interacts with MALAT1 and STAT3, inhibiting retinoblastoma cell proliferation and promoting apoptosis [30]. In chronic hypoxia-induced end-stage cardiovascular diseases, miR-20b-5p acts as an upstream regulator of HIF-1 in hypoxic H9C2 cells, mediating apoptosis via HIF-1 targeting and NF-κB pathway regulation [32]. Functional studies of Alzheimer’s disease have shown that miR-20b-5p targets RhoC, and inhibiting miR-20b-5p attenuates the β-amyloid-induced apoptosis of PC12 cells and reduces neuronal toxicity [33]. In inflammatory injuries caused by chronic hepatitis B, miR-20b-5p is a downstream factor of MALAT1 and interacts with MALAT1 to regulate TXNIP expression, modulating NLRP3 inflammasome activation and inflammatory responses [29]. In diabetic retinopathy, miR-20b-5p is downregulated in ARPE-19 cells exposed to high glucose. Increasing miR-20b-5p levels facilitates ARPE-19 cell proliferation and reduces apoptosis and pyroptosis, thereby mitigating diabetic retinopathy [34]. In human umbilical vein endothelial cells treated with high glucose, miR-20b-5p overexpression counteracts circHIPK3’s enhancement of angiogenesis, proliferation, and migration in diabetic ulcers [35]. Moreover, as a target of CircPTP4A2 in ischemic stroke, miR-20b-5p inhibition negates the effects of circPTP4A2 knockdown on promoting microglial M2 polarization [36]. In the context of mycobacterial infection, our study provides the first evidence that miR-20b-5p is downregulated during *M. bovis*-induced pyroptosis in THP-1 cells and that miR-20b-5p overexpression mitigates this process. This finding contrasts with the pro-pyroptotic role of miR-20b-5p reported in some other disease models [31,32] indicating that the functional outcome of miR-20b-5p modulation is highly context-dependent. Compared with *M. tuberculosis* infection—in which miR-20b-5p has not been extensively studied—our work identifies a previously unrecognized role for this miRNA specifically in *M. bovis* pathogenesis.

miRNAs regulate gene expression by binding to the 3’UTR or coding regions of target mRNAs, resulting in either cleavage or translational inhibition, affecting biological processes like immune responses [37]. P2RX7 was identified as a target gene of miR-20b-5p through whole-transcriptome sequencing and bioinformatics analysis. TargetScan predicted binding sites between miR-20b-5p and P2RX7, which were validated through dual-luciferase reporter assays and qPCR.

P2RX7, also known as P2X7R, is an essential member of the ATP purinergic receptor family, primarily functioning as an ATP-gated cation channel on immune cell surfaces. It plays a prominent role in initiating pro-inflammatory signaling in macrophages by regulating pro-inflammatory cytokine production and cell death [38]. It is also involved in various infectious and non-infectious diseases. High P2RX7 expression has been observed in a range of immune cells. In depression, P2RX7 activates the NLRP3 inflammasome, resulting in IL-1β release and depressive behavior in rats [39]. Additionally, P2RX7 modulates trichostatin, reducing uterine damage in mice exposed to tobacco smoke by promoting pyroptosis and apoptosis [40]. The P2X7 receptor is also implicated in migraine pathogenesis, where repeated inflammatory stimulation of the dura mater upregulates P2X7R, activating the NLRP3 inflammasome and releasing IL-1β and IL-18, leading to pyroptotic cell death. Pharmacological inhibition of P2X7R with Brilliant Blue G can alleviate cognitive impairment associated with dural inflammation [41]. Furthermore, P2RX7 mutations are linked to chronic non-bacterial osteomyelitis, as they reduce pyroptosis and extend cell survival [42]. During Trichinella infection, P2X7R activation in macrophages triggers the NLRP3 inflammasome, promoting IL-1β and IL-18 synthesis and secretion [43]. In the context of mycobacterial infection, P2RX7 mediates NLRP3 inflammasome activation in response to mycobacteria [44,45]. However, direct evidence linking P2RX7 to *M. bovis* virulent strain-induced pyroptosis is lacking. Our study fills this gap by demonstrating that P2RX7 is upregulated in *M. bovis*-infected THP-1 cells, and that its knockdown attenuates pyroptotic cell death. This finding aligns with the known pro-inflammatory function of P2RX7 in other infection models [43], suggesting that the P2RX7-mediated pyroptosis pathway is conserved between *M. tuberculosis* and *M. bovis*. Nevertheless, subtle differences in the kinetics or magnitude of P2RX7 activation between the two mycobacterial species cannot be ruled out, warranting further comparative investigation.

In summary, the MALAT1–miR-20b-5p–P2RX7 axis is implicated in *M. bovis*-induced pyroptosis of THP-1 cells. To the best of our knowledge, this is the first report of a ceRNA network involving MALAT1, miR-20b-5p, and P2RX7 specifically in the context of *M. bovis* infection. Studies have revealed that, in *M. bovis*-infected THP-1 cells, MALAT1 overexpression increases P2RX7 expression, which promotes pyroptosis. However, the upregulation of miR-20b-5p partially counteracts this effect, suggesting that MALAT1 may act as a competitive endogenous RNA, sequestering miR-20b-5p and modulating the expression of its target, P2RX7, which regulates *M. bovis*-mediated pyroptosis in THP-1 cells. Given the conserved host response mechanisms discussed above, we anticipate that this regulatory axis may also be operative in *M. tuberculosis* infection, although direct experimental validation is required. The novelty of our study lies not only in identifying this axis but also in providing the first comprehensive transcriptomic landscape of *M. bovis*-induced pyroptosis, thereby advancing our understanding of zoonotic TB pathogenesis at the molecular level.

Several limitations of this study should be mentioned. First, the relationship between miRNA and lncRNA is complex, and potential targets of MALAT1 beyond those identified may exist, requiring further investigation. Second, the mechanistic evidence is incomplete; for example, we did not elucidate the upstream signals by which *M. bovis* infection induces MALAT1 expression, nor did we perform direct RNA–RNA interaction assays (e.g., RIP or RNA pull-down) to validate the ceRNA mechanism. Moreover, the rescue experiments showed only partial reversal by miR-20b-5p, suggesting that additional regulatory molecules or parallel pathways contribute to P2RX7 modulation. Third, and most critically regarding the physiological relevance of our findings, all experiments were conducted exclusively in the PMA-differentiated THP-1 cell line, an immortalized human monocytic model. Although THP-1 cells are a widely accepted and well-characterized model for mechanistic studies of mycobacterial infection and pyroptosis and have been shown to exhibit functional similarities to primary human monocyte-derived macrophages in response to mycobacterial infection [46], we fully recognize that they do not fully recapitulate primary macrophage biology. Therefore, validation in primary macrophages (e.g., human peripheral blood-derived macrophages or bovine alveolar macrophages) is an essential and indispensable next step to confirm the translational relevance of the MALAT1–miR-20b-5p–P2RX7 axis identified in this study. Other members of our laboratory are already pursuing this by continuing to perform in-depth mining of the existing omics data and are planning to conduct mechanistic validation using primary cells and animal models as a follow-up to this study. Fourth, while we primarily assessed the roles and mechanisms of MALAT1, miR-20b-5p, and P2RX7 in *M. bovis*-mediated THP-1 pyroptosis, additional in vivo experiments are required for validation. Specifically, future studies using *M. bovis* infection models in cattle or suitable animal models (e.g., guinea pigs or rabbits) are needed to confirm the physiological relevance of the MALAT1–miR-20b-5p–P2RX7 axis. Fifth, several technical limitations should be acknowledged: (1) The GFP tag in both constructs may interfere with native MALAT1 function and P2RX7 localization/function, respectively, though P2RX7 core functionality was retained in our assays. We acknowledge these as clear limitations of the present study. (2) CCK-8 lacked matched transfection controls and orthogonal viability assays (e.g., Trypan blue exclusion); results reflect metabolic activity rather than direct cell number. (3) Given that this study is an exploratory mechanistic investigation primarily focused on trend identification and screening of key molecules, all experiments were performed with technical replicates only (three independent wells per group), rather than independent biological replicates. As such, future studies should validate the key findings using independent biological replicates. Despite these limitations, our study provides a crucial molecular framework for understanding *M. bovis*–host interactions and opens new avenues for therapeutic intervention against zoonotic TB.

## 5. Conclusions

This study established a pyroptosis model of THP-1 macrophages mediated by *M. bovis* infection. It characterized the expression profiles of mRNA, lncRNA, circRNA, and miRNA in THP-1 cells following *M. bovis* infection and constructed a proposed ceRNA regulatory network. The regulatory mechanisms of differentially expressed miR-20b-5p, MALAT1, and the target gene P2RX7 in *M. bovis*-mediated THP-1 pyroptosis were preliminarily explored. Specifically, *M. bovis* infection upregulated MALAT1 and P2RX7 expression and downregulated miR-20b-5p levels in THP-1 cells. Downregulation of MALAT1 or P2RX7 and overexpression of miR-20b-5p mitigated *M. bovis*-induced pyroptosis in THP-1 cells. Furthermore, miR-20b-5p was identified as a target of MALAT1 and was shown to target P2RX7. These findings suggest that the MALAT1–miR-20b-5p–P2RX7 regulatory axis may participate in *M. bovis*-induced pyroptosis in THP-1 cells, providing a preliminary molecular framework for further investigation.

## Data Availability

The data presented in this study are openly available. The original sequencing data of the omics reported in this paper have been deposited at the China National Center for Bioinformation/Beijing Institute of Genomics, Chinese Academy of Sciences (GSA: CRA026512 and CRA026519), and can be accessed at https://ngdc.cncb.ac.cn/gsa (accessed on 27 May 2026).

## Supplementary Materials

The following supporting information can be downloaded at: https://www.mdpi.com/article/10.3390/vetsci13060545/s1, Figure S1: Differentially expressed RNAs in THP-1 with *M. bovis* infection. (A-D) represent the volcano map and MA map of differentially expressed mRNAs, lncRNAs, circRNAs and miRNAs, respectively. Figure S2. GO enrichment analysis of differentially expressed RNAs in THP-1 infected by *M. bovis*. (A) GO enrichment of differentially expressed mRNAs. (B) GO enrichment results of differentially expressed lncRNA target genes. (C) GO enrichment results of differentially expressed circRNA source genes. (D) GO enrichment results of differentially expressed miRNA target genes. Figure S3. KEGG enrichment analysis of differentially expressed RNAs in THP-1 infected by M. bovis. (A) KEGG enrichment of differentially expressed mRNAs. (B) KEGG enrichment results of differentially expressed lncRNA target genes. (C) KEGG enrichment results of differentially expressed circRNA source genes. (D) KEGG enrichment results of differentially expressed miRNA target genes. Figure S4. Figure S4. Original flow cytometry plots for Figure 1D. Figure S5. Original gel electrophoresis images for Figure 4A and Figure 6A. Figure S6. Uncropped original western blot images for Figure 4H, Figure 5F and Figure 6H. Table S1: The primer information of qPCR. Table S2. Primers used for plasmid construction. Table S3. siRNA sequences. Table S4. Oligonucleotide sequences for dual-luciferase reporter assay. Table S5. Differentially expressed mRNAs, lncRNAs, miRNAs, and circRNAs. Table S6. lncRNAs used for ceRNA network construction. Table S7. miRNAs used for ceRNA network construction. Table S8. mRNAs used for ceRNA network construction.
