# Supplementary material for: MALAT1–miR-20b-5p–P2RX7 Axis Regulates Mycobacterium bovis-Induced THP-1 Pyroptosis
Source: Vet Sci. 2026 May 31;13(6):545. doi: 10.3390/vetsci13060545 (PMC13308039; doi:10.3390/vetsci13060545)
Supplement: Supplementary file 1 [file vetsci-13-00545-s001.zip › vetsci-4309857 Supplementary Materials.pdf]

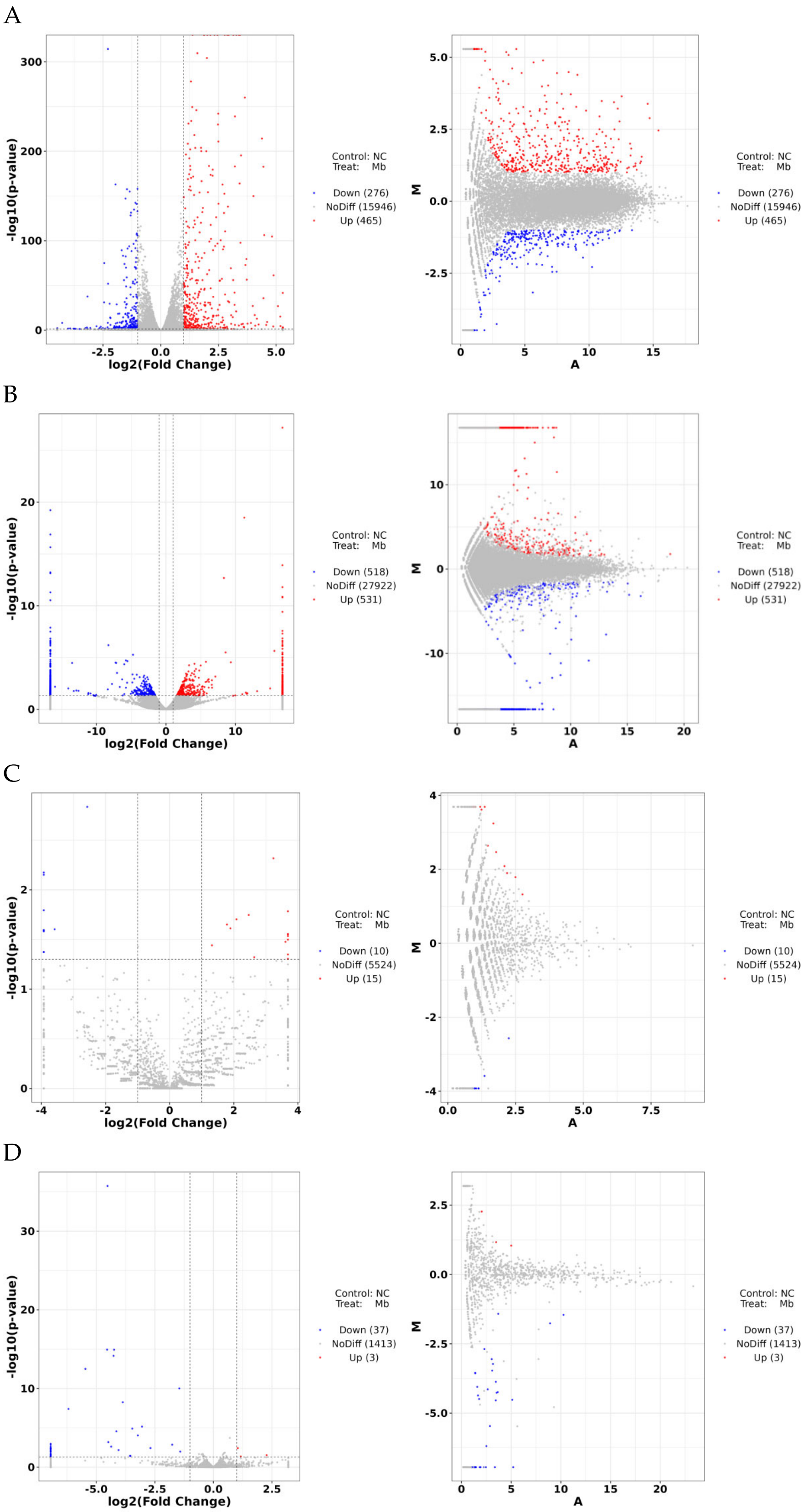

Figure S1. Differentially expressed RNAs in THP-1 with *M. bovis* infection. (A-D) represent the volcano map and MA map of differentially expressed mRNAs, lncRNAs, circRNAs and miRNAs, respectively.

A

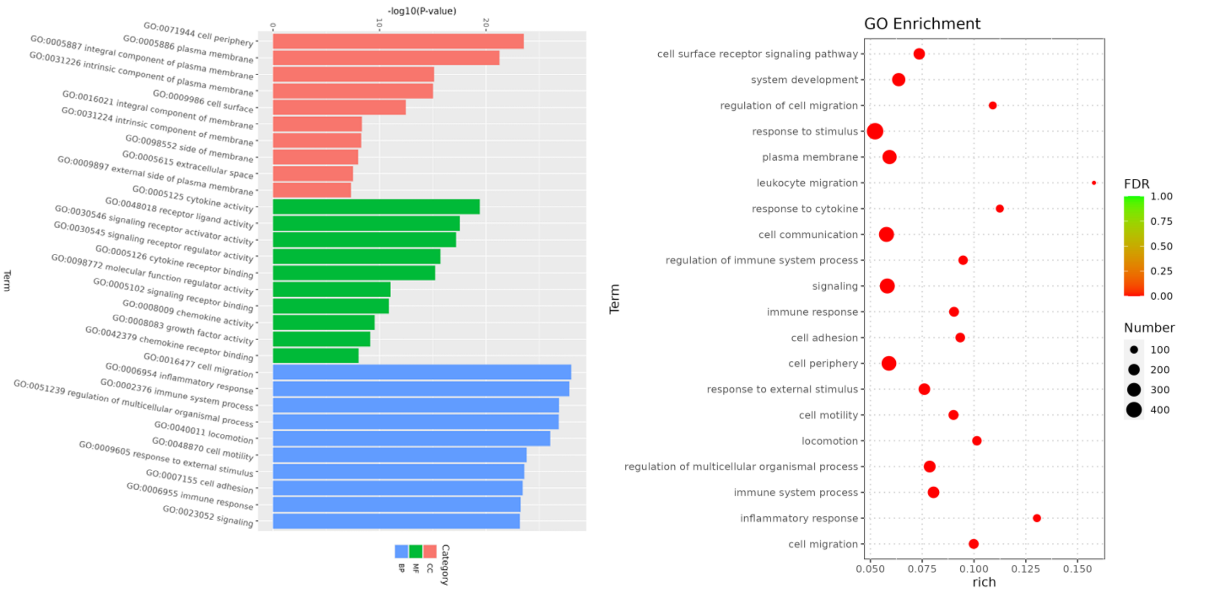
-log10(P-value)
0
10
20
Term
GO:0071944 cell periphery
GO:0005886 plasma membrane
GO:0005887 integral component of plasma membrane
GO:0031226 intrinsic component of plasma membrane
GO:0009986 cell surface
GO:0016021 integral component of membrane
GO:0031224 intrinsic component of membrane
GO:0098552 side of membrane
GO:0005615 extracellular space
GO:0009897 external side of plasma membrane
GO:0005125 cytokine activity
GO:0048018 receptor ligand activity
GO:0030546 signaling receptor activator activity
GO:0030545 signaling receptor regulator activity
GO:0005126 cytokine receptor binding
GO:0098772 molecular function regulator activity
GO:0005102 signaling receptor binding
GO:0008009 chemokine activity
GO:0008083 growth factor activity
GO:0042379 chemokine receptor binding
GO:0016477 cell migration
GO:0006954 inflammatory response
GO:0002376 immune system process
GO:0051239 regulation of multicellular organismal process
GO:0040011 locomotion
GO:0048870 cell motility
GO:0009605 response to external stimulus
GO:0007155 cell adhesion
GO:0006955 immune response
GO:0023052 signaling
Category
BP
MF
CC
GO Enrichment
cell surface receptor signaling pathway
system development
regulation of cell migration
response to stimulus
plasma membrane
leukocyte migration
response to cytokine
cell communication
regulation of immune system process
signaling
immune response
cell adhesion
cell periphery
response to external stimulus
cell motility
locomotion
regulation of multicellular organismal process
immune system process
inflammatory response
cell migration
Term
0.050
0.075
0.100
0.125
0.150
rich
FDR
1.00
0.75
0.50
0.25
0.00
Number
100
200
300
400

B

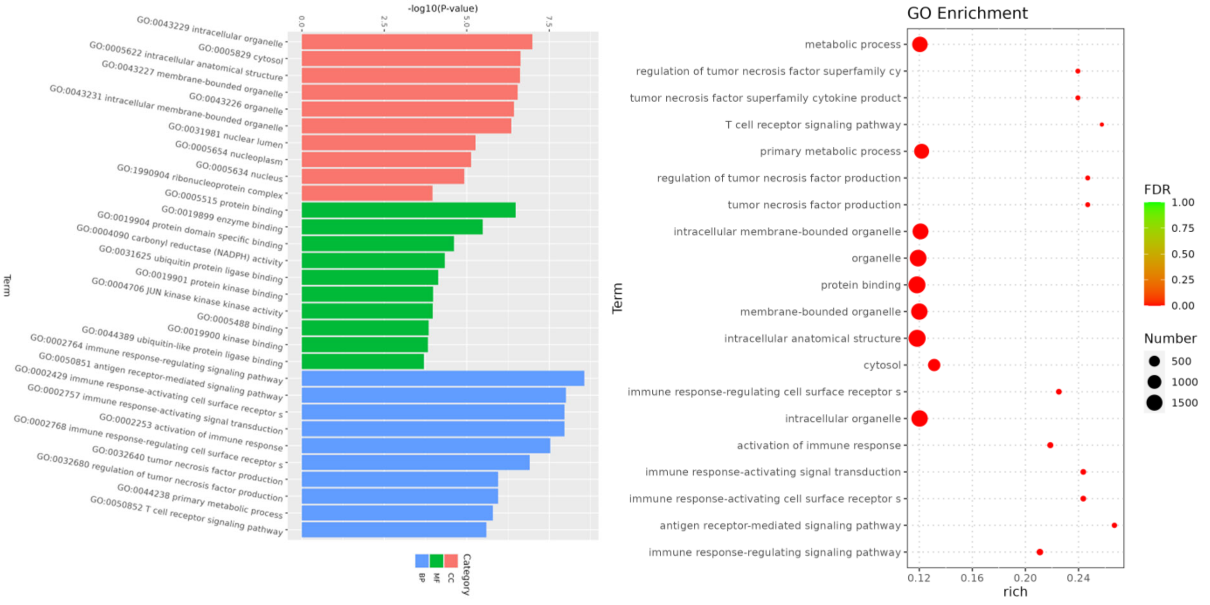
-log10(P-value)
0.0
2.5
5.0
7.5
Term
GO:0043229 intracellular organelle
GO:0005829 cytosol
GO:0005622 intracellular anatomical structure
GO:0043227 membrane-bounded organelle
GO:0043226 organelle
GO:0043231 intracellular membrane-bounded organelle
GO:0031981 nuclear lumen
GO:0005654 nucleoplasm
GO:0005634 nucleus
GO:1990904 ribonucleoprotein complex
GO:0005515 protein binding
GO:0019899 enzyme binding
GO:0019904 protein domain specific binding
GO:0004090 carbonyl reductase (NADPH) activity
GO:0031625 ubiquitin protein ligase binding
GO:0019901 protein kinase binding
GO:0004706 JUN kinase kinase kinase activity
GO:0005488 binding
GO:0019900 kinase binding
GO:0044389 ubiquitin-like protein ligase binding
GO:0002764 immune response-regulating signaling pathway
GO:0050851 antigen receptor-mediated signaling pathway
GO:0002429 immune response-activating cell surface receptor s
GO:0002757 immune response-activating signal transduction
GO:0002253 activation of immune response
GO:0002768 immune response-regulating cell surface receptor s
GO:0032640 tumor necrosis factor production
GO:0032680 regulation of tumor necrosis factor production
GO:0044238 primary metabolic process
GO:0050852 T cell receptor signaling pathway
Category
BP
MF
CC
GO Enrichment
metabolic process
regulation of tumor necrosis factor superfamily cy
tumor necrosis factor superfamily cytokine product
T cell receptor signaling pathway
primary metabolic process
regulation of tumor necrosis factor production
tumor necrosis factor production
intracellular membrane-bounded organelle
organelle
protein binding
membrane-bounded organelle
intracellular anatomical structure
cytosol
immune response-regulating cell surface receptor s
intracellular organelle
activation of immune response
immune response-activating signal transduction
immune response-activating cell surface receptor s
antigen receptor-mediated signaling pathway
immune response-regulating signaling pathway
Term
0.12
0.16
0.20
0.24
rich
FDR
1.00
0.75
0.50
0.25
0.00
Number
500
1000
1500

C

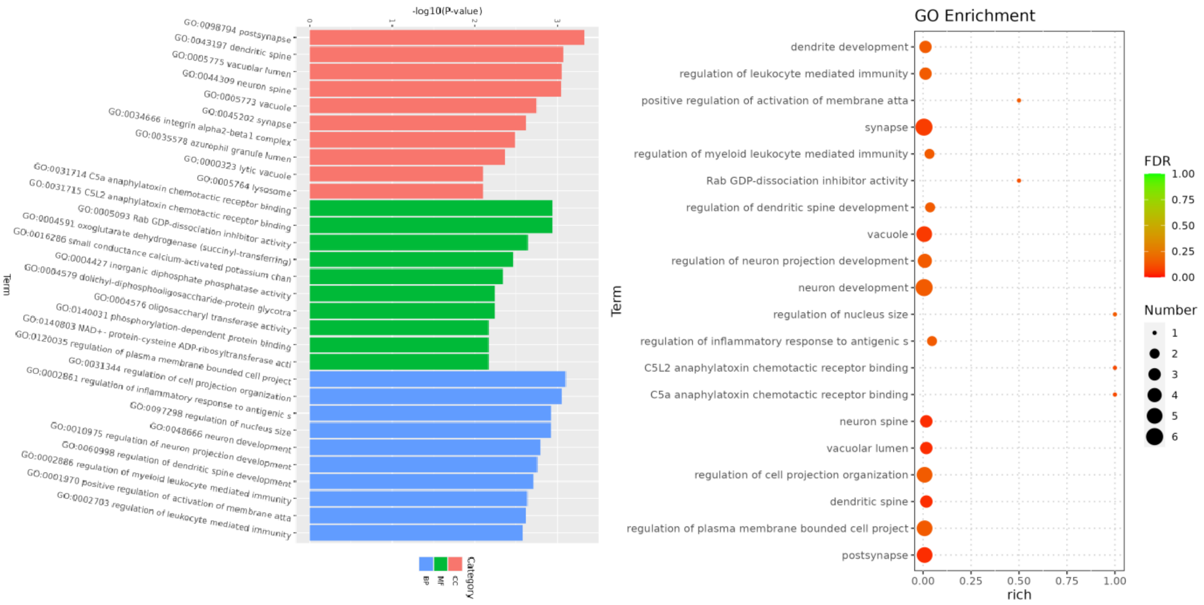

D

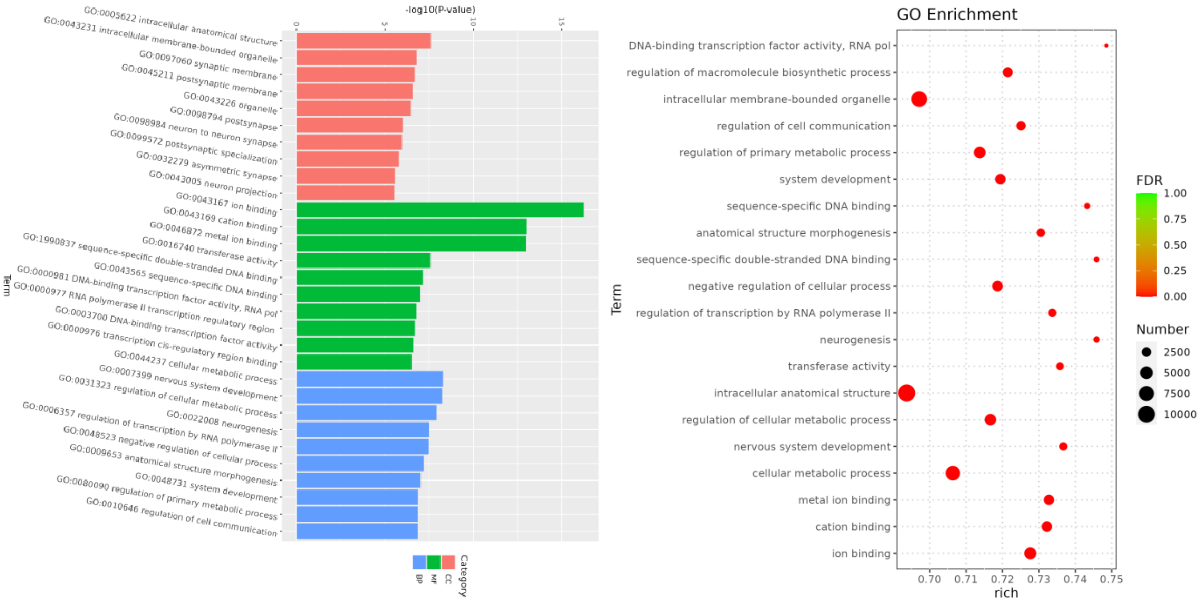

Figure S2. GO enrichment analysis of differentially expressed RNAs in THP-1 infected by *M. bovis*. (A) GO enrichment of differentially expressed mRNAs. (B) GO enrichment results of differentially expressed lncRNA target genes. (C) GO enrichment results of differentially expressed circRNA source genes. (D) GO enrichment results of differentially expressed miRNA target genes.

A

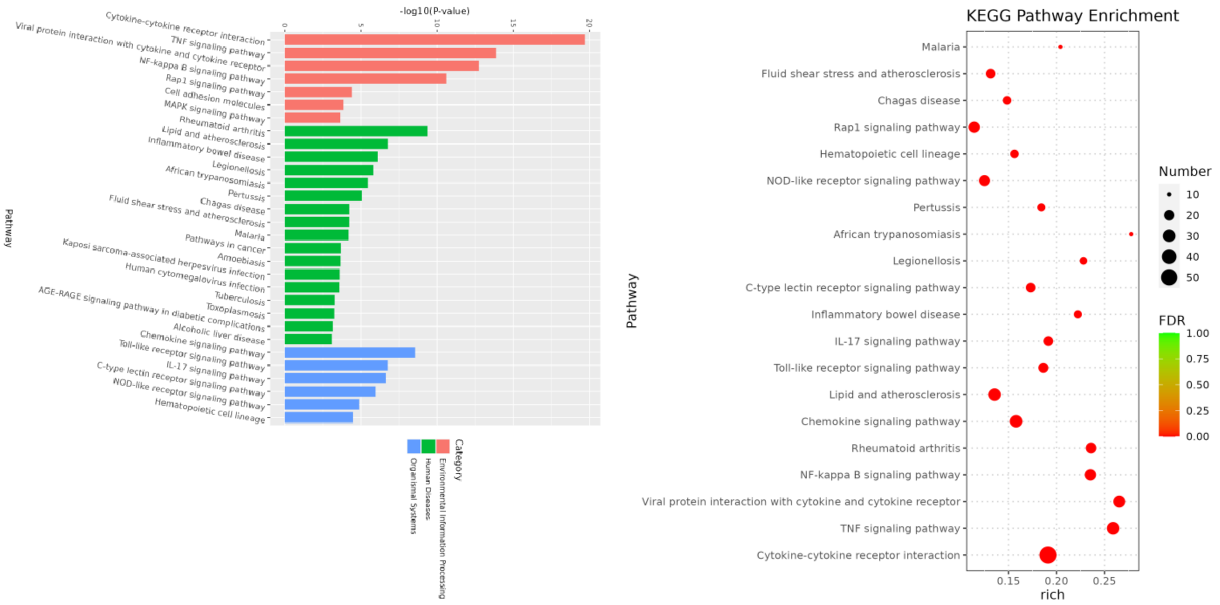
-log10(P-value)
Pathway
Cytokine-cytokine receptor interaction
TNF signaling pathway
Viral protein interaction with cytokine and cytokine receptor
NF-kappa B signaling pathway
Rap1 signaling pathway
Cell adhesion molecules
MAPK signaling pathway
Rheumatoid arthritis
Lipid and atherosclerosis
Inflammatory bowel disease
Legionellosis
African trypanosomiasis
Pertussis
Chagas disease
Fluid shear stress and atherosclerosis
Malaria
Pathways in cancer
Amoebiasis
Kaposi sarcoma-associated herpesvirus infection
Human cytomegalovirus infection
Tuberculosis
Toxoplasmosis
AGE-RAGE signaling pathway in diabetic complications
Alcoholic liver disease
Chemokine signaling pathway
Toll-like receptor signaling pathway
IL-17 signaling pathway
C-type lectin receptor signaling pathway
NOD-like receptor signaling pathway
Hematopoietic cell lineage
Category
Environmental Information Processing
Human Diseases
Organismal Systems
KEGG Pathway Enrichment
Malaria
Fluid shear stress and atherosclerosis
Chagas disease
Rap1 signaling pathway
Hematopoietic cell lineage
NOD-like receptor signaling pathway
Pertussis
African trypanosomiasis
Legionellosis
C-type lectin receptor signaling pathway
Inflammatory bowel disease
IL-17 signaling pathway
Toll-like receptor signaling pathway
Lipid and atherosclerosis
Chemokine signaling pathway
Rheumatoid arthritis
NF-kappa B signaling pathway
Viral protein interaction with cytokine and cytokine receptor
TNF signaling pathway
Cytokine-cytokine receptor interaction
Pathway
rich
0.15
0.20
0.25
Number
10
20
30
40
50
FDR
1.00
0.75
0.50
0.25
0.00

B

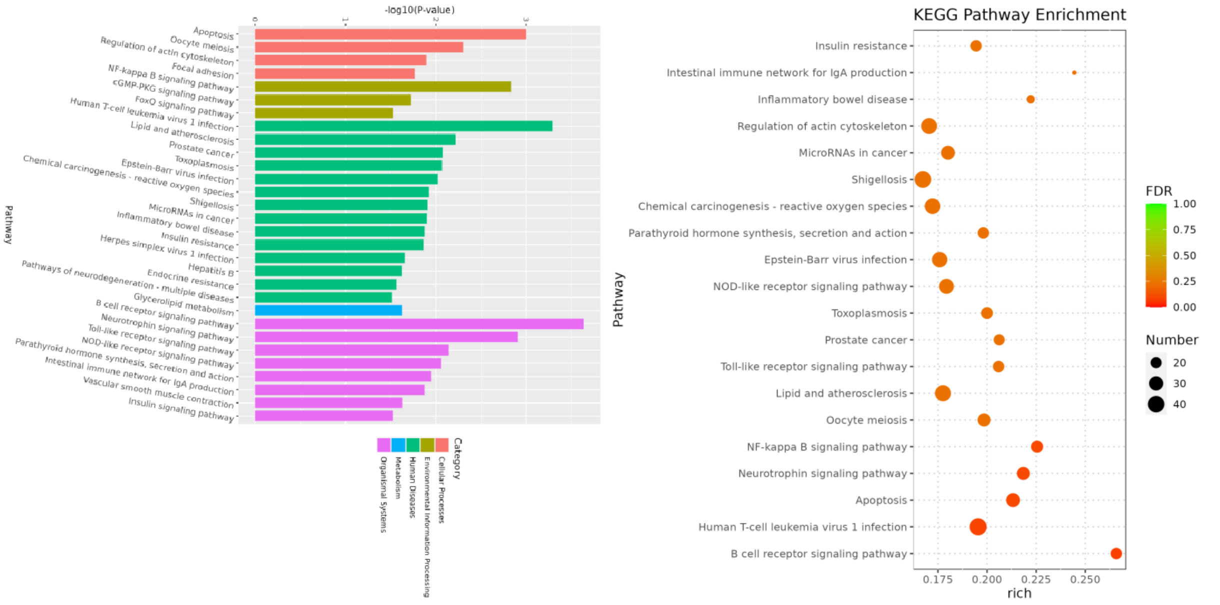
-log10(P-value)
Pathway
Apoptosis
Oocyte meiosis
Regulation of actin cytoskeleton
Focal adhesion
NF-kappa B signaling pathway
cGMP-PKG signaling pathway
FoxO signaling pathway
Human T-cell leukemia virus 1 infection
Lipid and atherosclerosis
Prostate cancer
Toxoplasmosis
Epstein-Barr virus infection
Chemical carcinogenesis - reactive oxygen species
Shigellosis
MicroRNAs in cancer
Inflammatory bowel disease
Insulin resistance
Herpes simplex virus 1 infection
Hepatitis B
Endocrine resistance
Pathways of neurodegeneration - multiple diseases
Glycerolipid metabolism
B cell receptor signaling pathway
Neurotrophin signaling pathway
Toll-like receptor signaling pathway
NOD-like receptor signaling pathway
Parathyroid hormone synthesis, secretion and action
Intestinal immune network for IgA production
Vascular smooth muscle contraction
Insulin signaling pathway
Category
Cellular Processes
Environmental Information Processing
Human Diseases
Metabolism
Organismal Systems
KEGG Pathway Enrichment
Insulin resistance
Intestinal immune network for IgA production
Inflammatory bowel disease
Regulation of actin cytoskeleton
MicroRNAs in cancer
Shigellosis
Chemical carcinogenesis - reactive oxygen species
Parathyroid hormone synthesis, secretion and action
Epstein-Barr virus infection
NOD-like receptor signaling pathway
Toxoplasmosis
Prostate cancer
Toll-like receptor signaling pathway
Lipid and atherosclerosis
Oocyte meiosis
NF-kappa B signaling pathway
Neurotrophin signaling pathway
Apoptosis
Human T-cell leukemia virus 1 infection
B cell receptor signaling pathway
Pathway
rich
0.175
0.200
0.225
0.250
FDR
1.00
0.75
0.50
0.25
0.00
Number
20
30
40

C

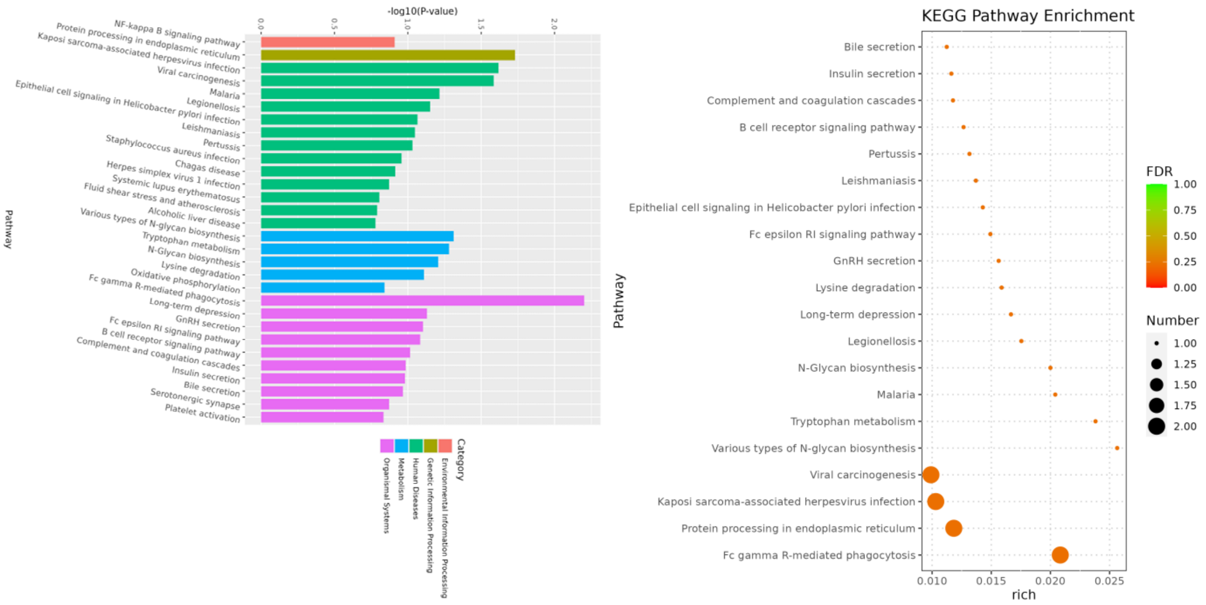

D

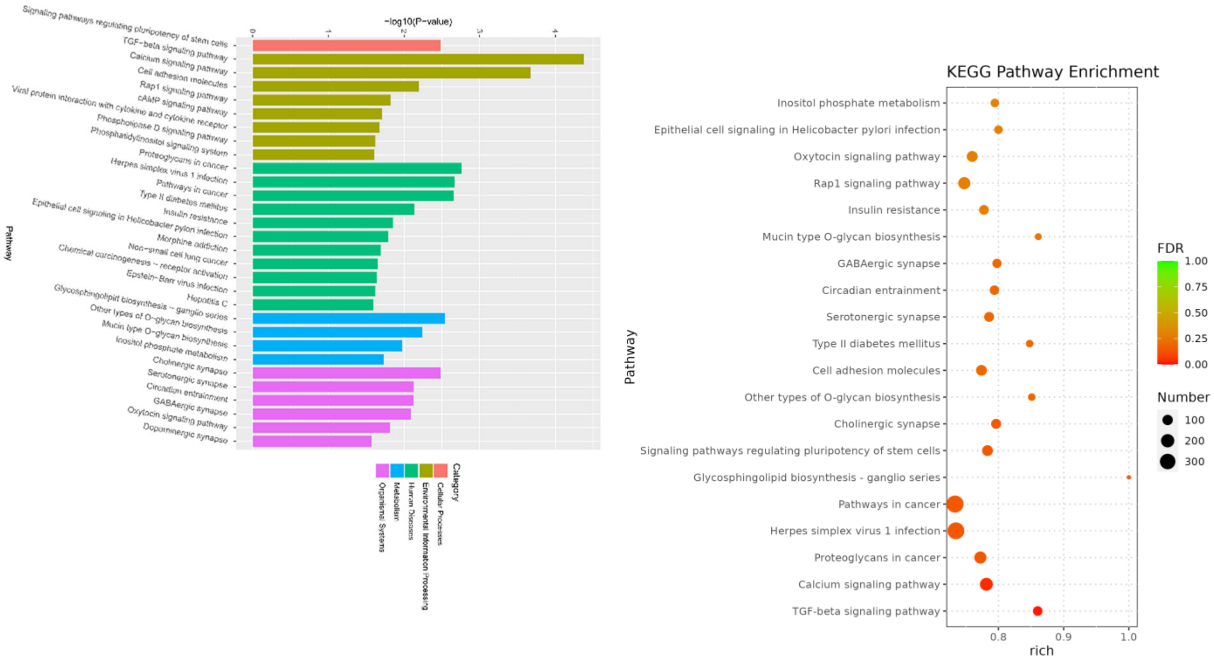

Figure S3. KEGG enrichment analysis of differentially expressed RNAs in THP-1 infected by *M. bovis*. (A) KEGG enrichment of differentially expressed mRNAs. (B) KEGG enrichment results of differentially expressed lncRNA target genes. (C) KEGG enrichment results of differentially expressed circRNA source genes. (D) KEGG enrichment results of differentially expressed miRNA target genes.

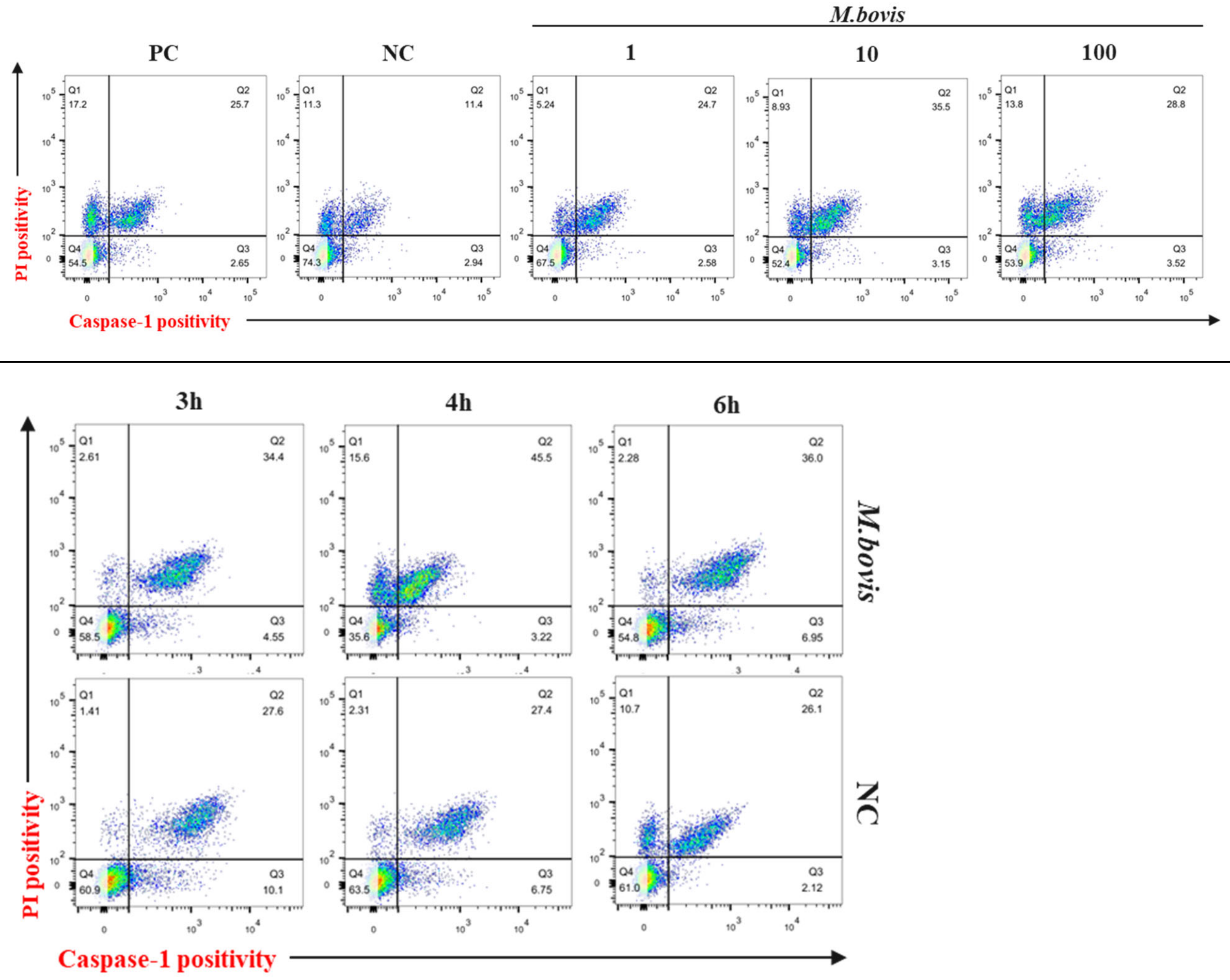

Figure S4. Original flow cytometry plots for Figure 1D.

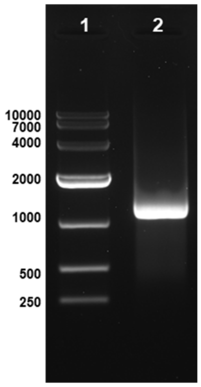

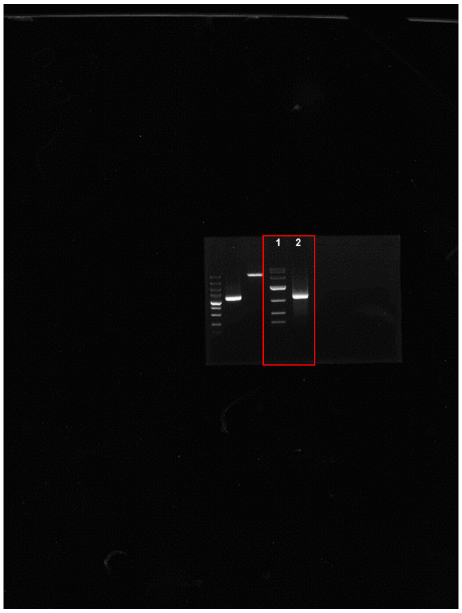

Manuscipt: MALAT1 gene amplification (1: DL10000, 2: MALAT1)	Orginal image: the original image of MALAT1 gene amplification was marked with a red box (1: DL10000, 2: MALAT1)
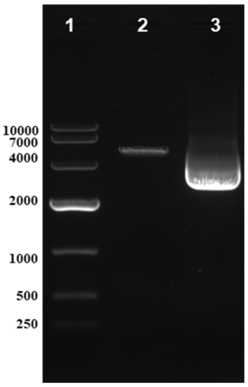	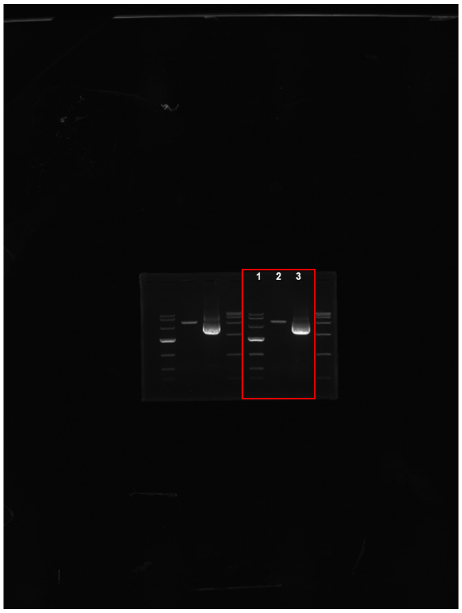
Manuscipt: double-enzyme digestion and identification of the pEGFP-C1 vector (1: DL10000, 2: digested pEGFP-C1, 3: pEGFP-C1 plasmid)	Orginal image: the original image of double-enzyme digestion and identification of the pEGFP-C1 vector was marked with a red box (1: DL10000, 2: digested pEGFP-C1, 3: pEGFP-C1 plasmid)
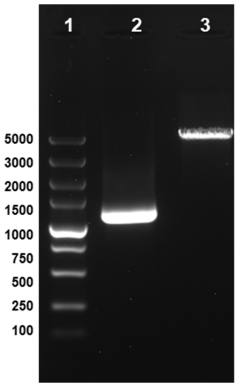	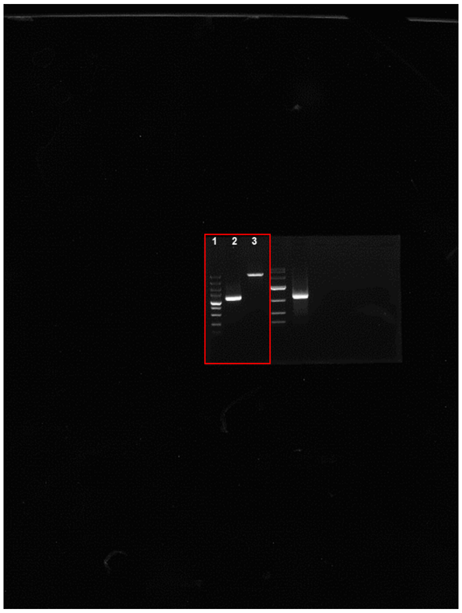
Manuscipt: identification of purified pEGFP-C1 and MALAT1 products (1: DL5000, 2: MALAT1, 3: pEGFP-C1)	Orginal image: the original image of identification of purified pEGFP-C1 and MALAT1 products was marked with a red box (1: DL5000, 2: MALAT1, 3: pEGFP-C1)

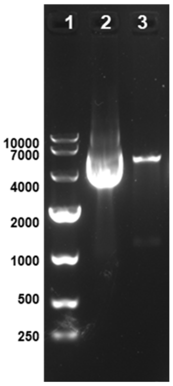	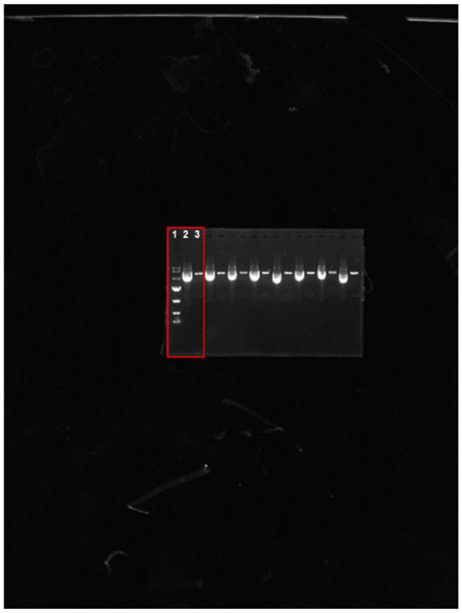
Manuscipt: the double-enzyme digestion of the endotoxin-free pEGFP-C1-MALAT1 plasmid (1: DL10000, 2: pEGFP-C1-MALAT1, 3: digested product)	Orginal image: the original image of double-enzyme digestion of the endotoxin-free pEGFP-C1-MALAT1 plasmid was marked with a red box (1: DL10000, 2: pEGFP-C1-MALAT1, 3: digested product)
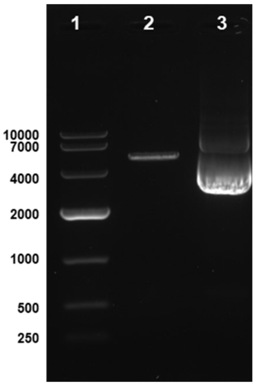	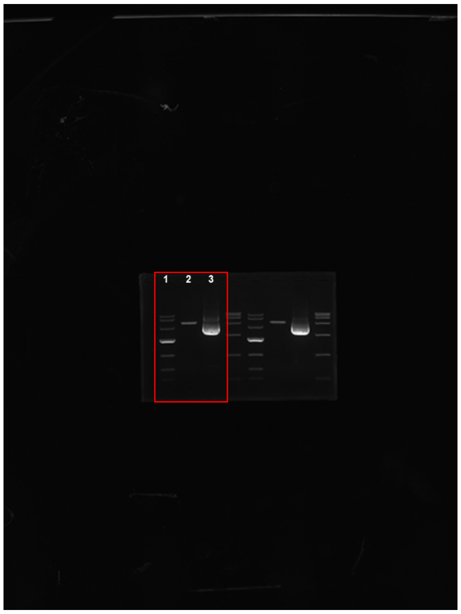
Manuscipt: double-enzyme digestion and identification of vector pEGFP-N2 (1: DL10000, 2: double digestion product, 3: pEGFP-N2 plasmid)	Orginal image: the original image of double-enzyme digestion and identification of vector pEGFP-N2 was marked with a red box (1: DL10000, 2: double digestion product, 3: pEGFP-N2 plasmid)

<table>
<tr>
<td>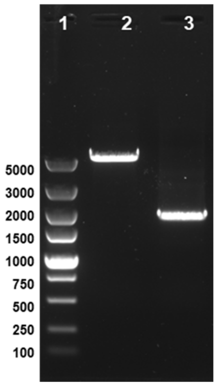
</td>
<td>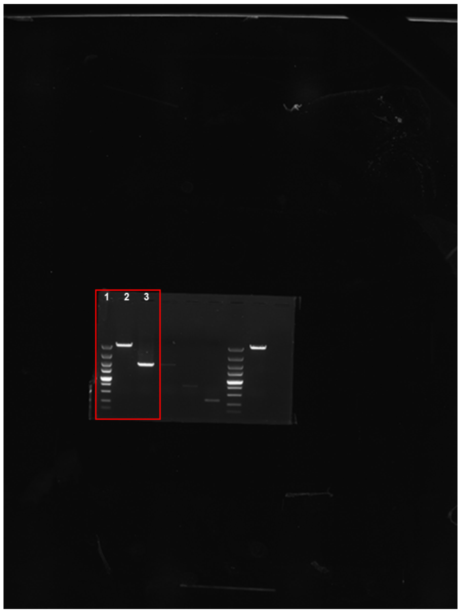
</td>
</tr>
<tr>
<td>Manuscipt: identification of purified pEGFP-N2 and P2RX7 products (1: DL5000, 2: pEGFP-N2, 3: P2RX7)</td>
<td>Orginal image: the original image of identification of purified pEGFP-N2 and P2RX7 products was marked with a red box (1: DL5000, 2: pEGFP-N2, 3: P2RX7)</td>
</tr>
<tr>
<td>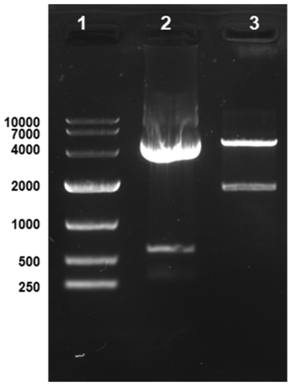
</td>
<td>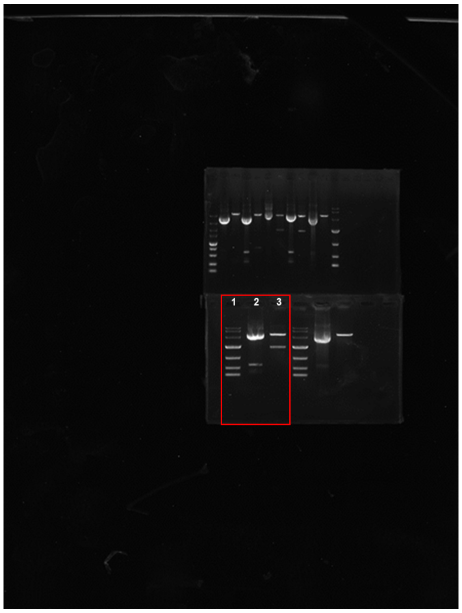
</td>
</tr>
<tr>
<td>Manuscipt: double digestion of the endotoxin-free pEGFP-N2-P2RX7 plasmid (1: DL10000, 2: pEGFP-N2-P2RX7 plasmid, 3: double digestion product)</td>
<td>Orginal image: the original image of double digested pEGFP-N2-P2RX7 plasmid without endotoxin was marked with a red box (1: DL10000, 2: pEGFP-N2-P2RX7 plasmid, 3: double digestion product)</td>
</tr>
<tr>
<td colspan="2">Figure S5. Original gel electrophoresis images for Figures 4A and 6A.</td>
</tr>
</table>

<table>
<tr>
<td>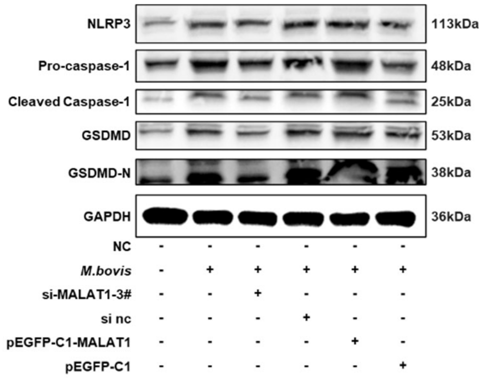
</td>
<td>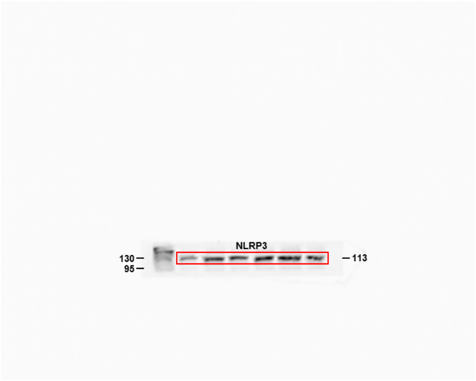
</td>
</tr>
<tr>
<td>Manuscipt: the effect of MALAT1 expression on NLRP3 protein level</td>
<td>Orginal image: the original image of the effect of MALAT1 expression on NLRP3 protein level was labeled with a red box and contained the protein name (NLRP3), molecular weight (113kDa) and protein marker (95-130kDa)</td>
</tr>
<tr>
<td>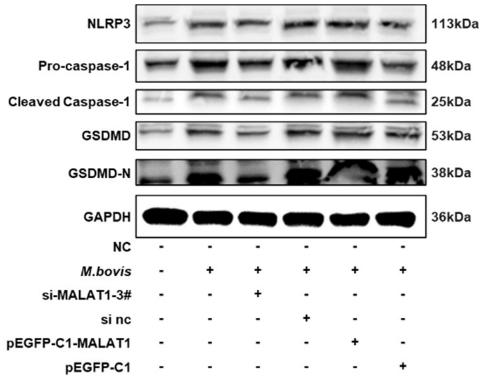
</td>
<td>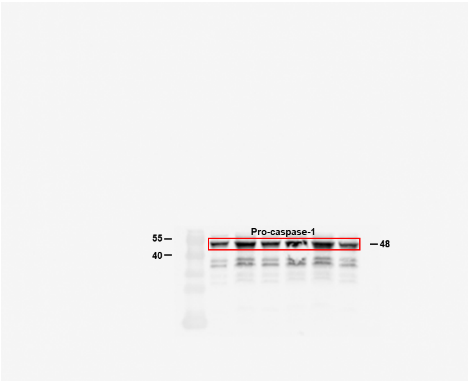
</td>
</tr>
<tr>
<td>Manuscipt: the effect of MALAT1 expression on pro-caspase-1 protein level</td>
<td>Orginal image: the original image of the effect of MALAT1 expression on pro-caspase-1 protein level was labeled with a red box and contained the protein name (pro-caspase-1), molecular weight (48kDa) and protein marker (40-55kDa)</td>
</tr>
<tr>
<td>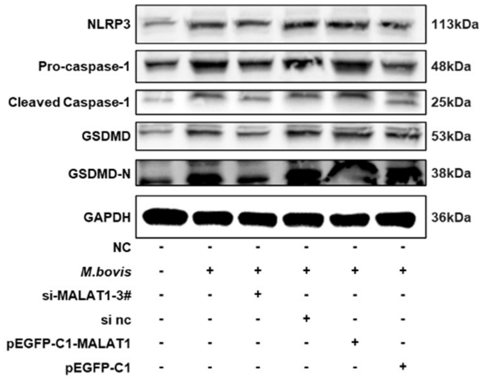
</td>
<td>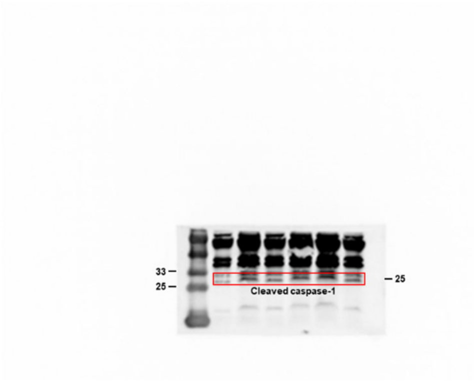
</td>
</tr>
<tr>
<td>Manuscipt: the effect of MALAT1 expression on cleaved caspase-1 protein level</td>
<td>Orginal image: the original image of the effect of MALAT1 expression on cleaved caspase-1 protein level was labeled with a red box and contained the protein name (cleaved caspase-1), molecular weight</td>
</tr>
</table>

<table>
<tr><td></td><td>(25kDa) and protein marker (25-33kDa)</td></tr>
<tr><td>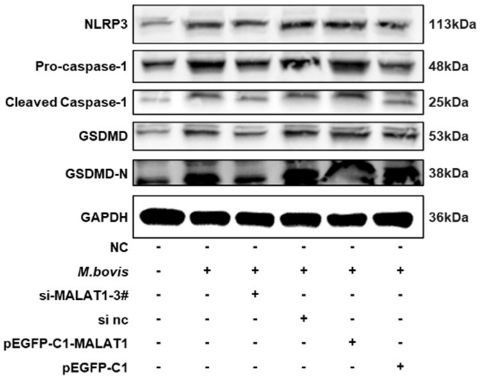
</td><td>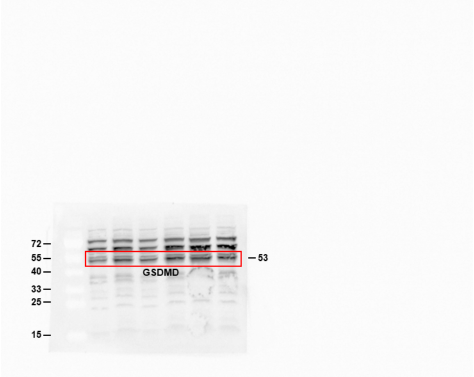
</td></tr>
<tr><td>Manuscipt: the effect of MALAT1 expression on GSDMD protein level</td><td>Orginal image: the original image of the effect of MALAT1 expression on GSDMD protein level was labeled with a red box and contained the protein name (GSDMD), molecular weight (53kDa) and protein marker (40-55kDa)</td></tr>
<tr><td>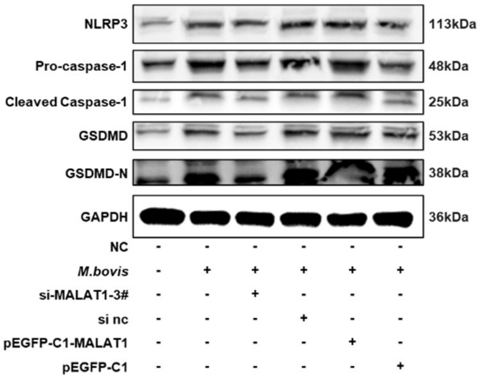
</td><td>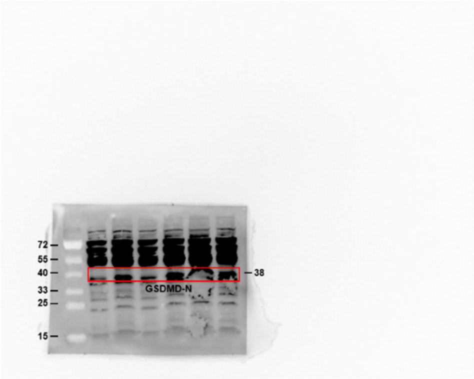
</td></tr>
<tr><td>Manuscipt: the effect of MALAT1 expression on GSDMD-N protein level</td><td>Orginal image: the original image of the effect of MALAT1 expression on GSDMD-N protein level was labeled with a red box and contained the protein name (GSDMD-N), molecular weight (38kDa) and protein marker (33-40kDa)</td></tr>
<tr><td>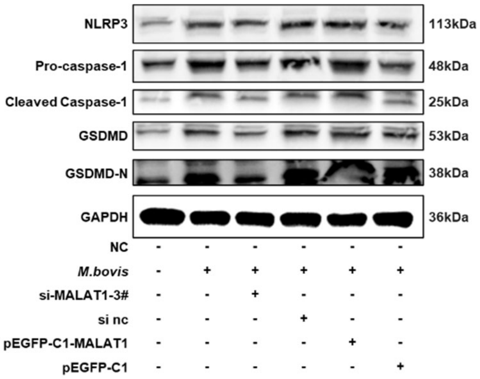
</td><td>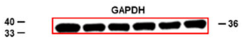
</td></tr>
<tr><td>Manuscipt: the effect of MALAT1 expression on GAPDH protein level</td><td>Orginal image: the original image of the effect of MALAT1 expression on GAPDH protein level was</td></tr>
</table>

<table>
<tr><td></td><td>labeled with a red box and contained the protein name (GAPDH), molecular weight (36kDa) and protein marker (33-40kDa)</td></tr>
<tr><td>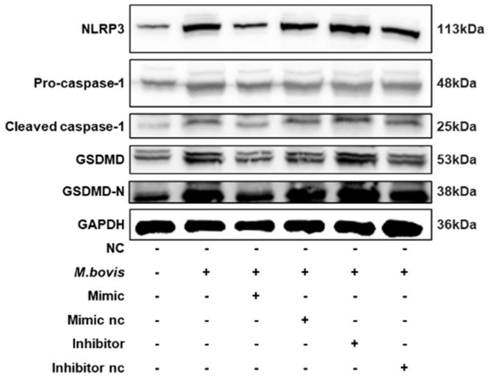
</td><td>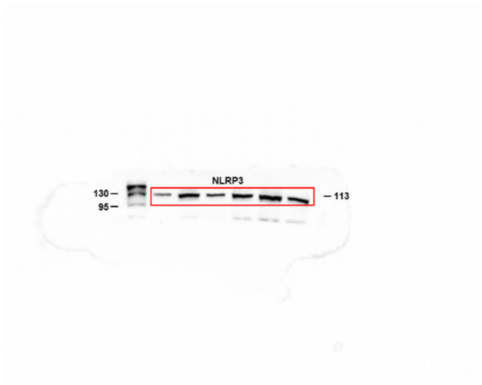
</td></tr>
<tr><td>Manuscipt: the influence of miR-20b-5p expression on NLRP3 protein level</td><td>Orginal image: the original image of the influence of miR-20b-5p expression on NLRP3 protein level was labeled with a red box and contained the protein name (NLRP3), molecular weight (113kDa) and protein marker (95-130kDa)</td></tr>
<tr><td>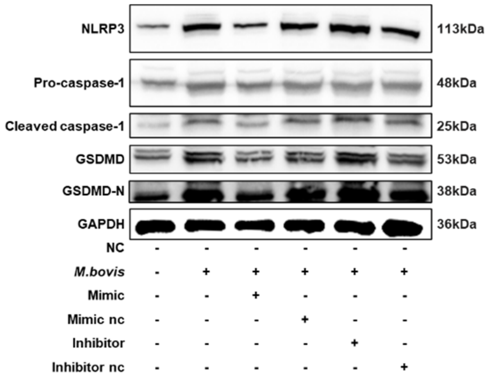
</td><td>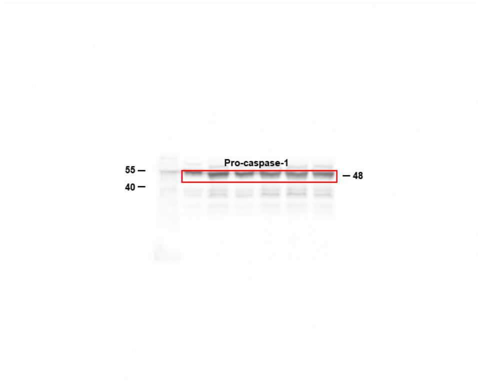
</td></tr>
<tr><td>Manuscipt: the influence of miR-20b-5p expression on pro-caspase-1 protein level</td><td>Orginal image: the original image of the influence of miR-20b-5p expression on pro-caspase-1 protein level was labeled with a red box and contained the protein name (pro-caspase-1), molecular weight (48kDa) and protein marker (40-55kDa)</td></tr>
<tr><td>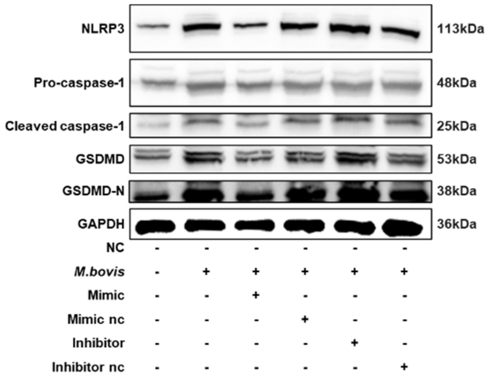
</td><td>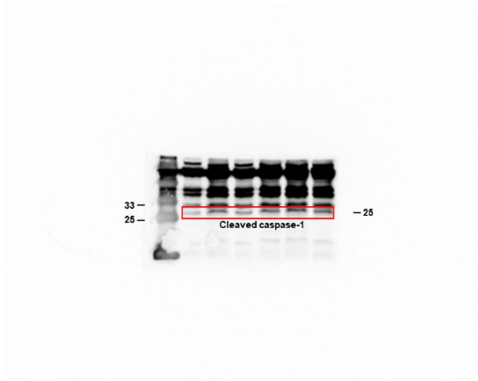
</td></tr>
</table>

<table>
<tr>
<td>Manuscipt: the influence of miR-20b-5p expression on cleaved caspase-1 protein level</td>
<td>Orginal image: the original image of the influence of miR-20b-5p expression on cleaved caspase-1 protein level was labeled with a red box and contained the protein name (cleaved caspase-1), molecular weight (25kDa) and protein marker (25-33kDa)</td>
</tr>
<tr>
<td>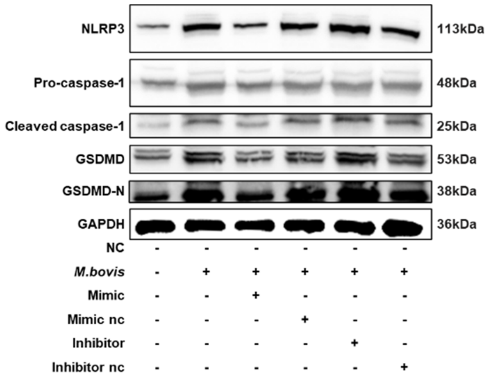
</td>
<td>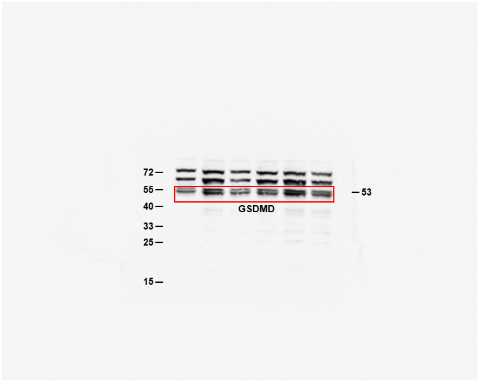
</td>
</tr>
<tr>
<td>Manuscipt: the influence of miR-20b-5p expression on GSDMD protein level</td>
<td>Orginal image: the original image of the influence of miR-20b-5p expression on GSDMD protein level was labeled with a red box and contained the protein name (GSDMD), molecular weight (53kDa) and protein marker (40-55kDa)</td>
</tr>
<tr>
<td>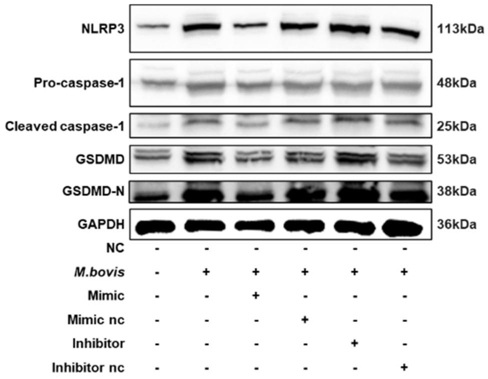
</td>
<td>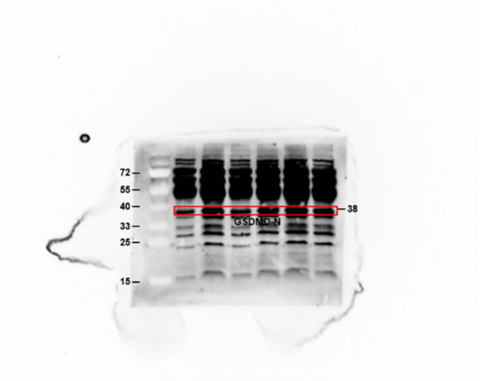
</td>
</tr>
<tr>
<td>Manuscipt: the influence of miR-20b-5p expression on GSDMD-N protein level</td>
<td>Orginal image: the original image of the influence of miR-20b-5p expression on GSDMD-N protein level was labeled with a red box and contained the protein name (GSDMD-N), molecular weight (38kDa) and protein marker (33-40kDa)</td>
</tr>
</table>

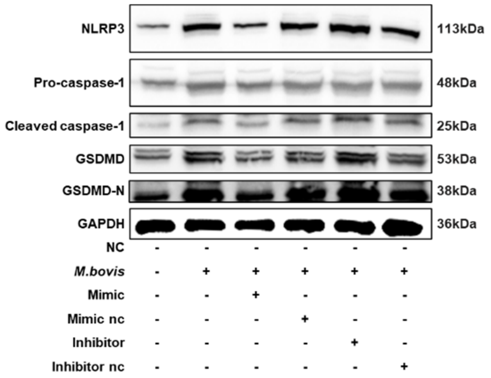	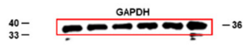
Manuscipt: the influence of miR-20b-5p expression on GAPDH protein level	Orginal image: the original image of the influence of miR-20b-5p expression on GAPDH protein level was labeled with a red box and contained the protein name (GAPDH), molecular weight (36kDa) and protein marker (33-40kDa)
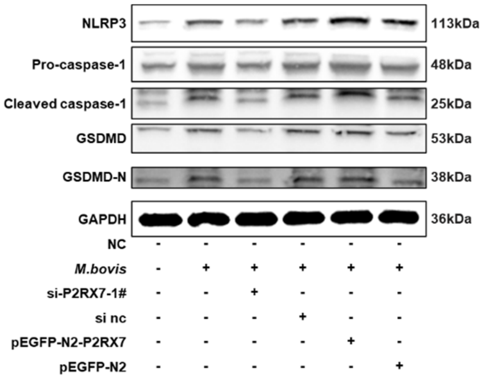	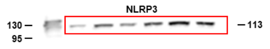
Manuscipt: the effect of P2RX7 expression on NLRP3 protein level	Orginal image: the original image of the effect of P2RX7 expression on NLRP3 protein level was labeled with a red box and contained the protein name (NLRP3), molecular weight (113kDa) and protein marker (95-130kDa)
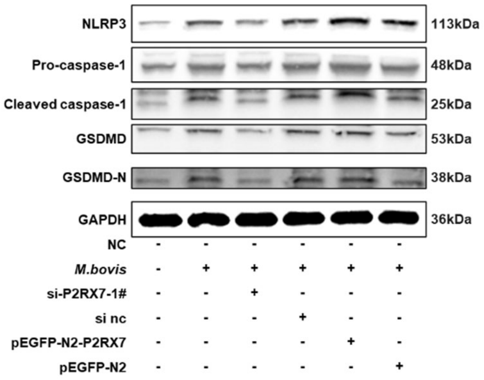	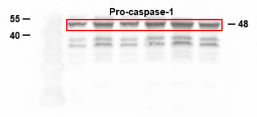
Manuscipt: the effect of P2RX7 expression on pro-caspase-1 protein level	Orginal image: the original image of the effect of P2RX7 expression on pro-caspase-1 protein level was labeled with a red box and contained the protein name (pro-caspase-1), molecular weight

	(48kDa) and protein marker (40-55kDa)
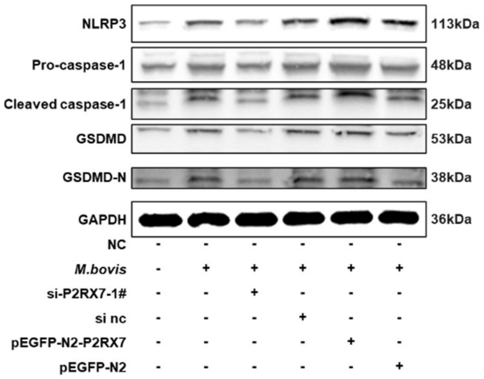	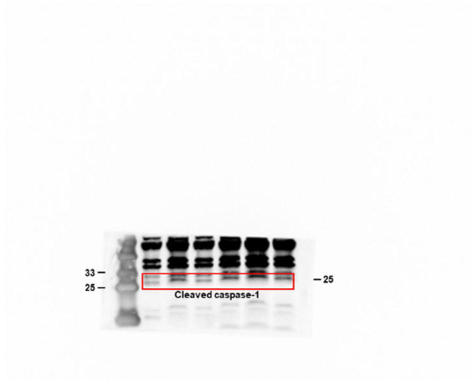
Manuscipt: the effect of P2RX7 expression on cleaved caspase-1 protein level	Orginal image: the original image of the effect of P2RX7 expression on cleaved caspase-1 protein level was labeled with a red box and contained the protein name (cleaved caspase-1), molecular weight (25kDa) and protein marker (25-33kDa)
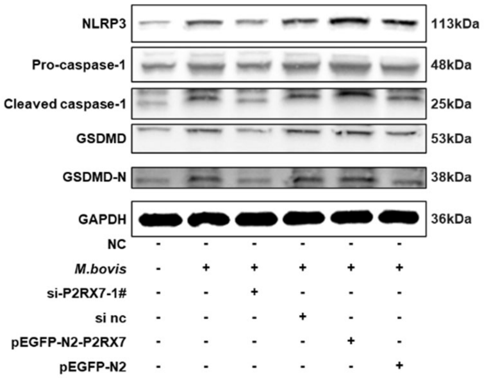	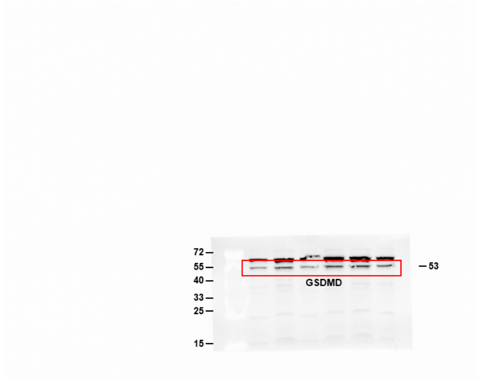
Manuscipt: the effect of P2RX7 expression on GSDMD protein level	Orginal image: the original image of the effect of P2RX7 expression on GSDMD protein level was labeled with a red box and contained the protein name (GSDMD), molecular weight (53kDa) and protein marker (40-55kDa)
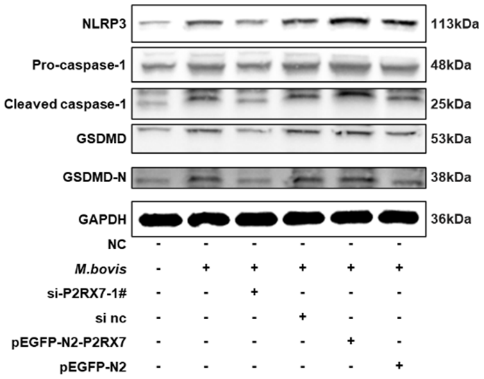	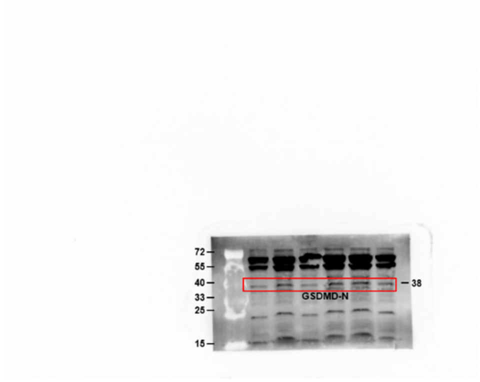
Manuscipt: the effect of P2RX7 expression on GSDMD-N protein level	Orginal image: the original image of the effect of P2RX7 expression on GSDMD-N protein level was

<table>
<tr><td></td><td>labeled with a red box and contained the protein name (GSDMD-N), molecular weight (38kDa) and protein marker (33-40kDa)</td></tr>
<tr><td>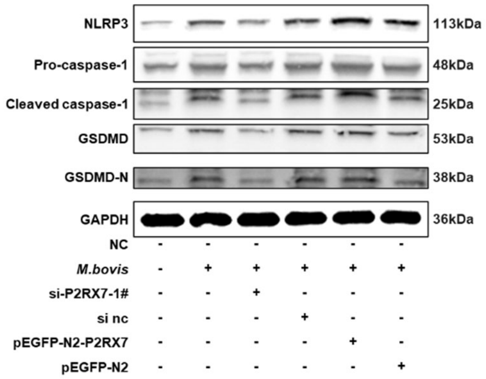
</td><td>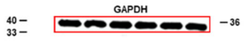
</td></tr>
<tr><td>Manuscipt: the effect of P2RX7 expression on GAPDH protein level</td><td>Orginal image: the original image of the effect of P2RX7 expression on GAPDH protein level was labeled with a red box and contained the protein name (GAPDH), molecular weight (36kDa) and protein marker (33-40kDa)</td></tr>
<tr><td colspan="2">Figure S6. Uncropped original western blot images for Figures 4H, 5F, and 6H.</td></tr>
</table>

Table S1. The primers used for qPCR

Target genes	Primer names	Sequence (5′-3′)
NLRP3	NLRP3-F	CGTGAGTCCCATTAAGATGGAGT
	NLRP3-R	CCCGACAGTGGATATAGAACAGA
pro-caspase-1	Caspase-1-F	TTTCCGCAAGGTTCGATTTTCA
	Caspase-1-R	GGCATCTGCGCTCTACCATC
GSDMD	GSDMD-F	GCCTCCACAACTTCCTGACAGATG
	GSDMD-R	GGTCTCCACCTCTGCCCGTAG
IL12B	IL12B-F	GACCTTGGACCAGAGCAGTGAG
	IL12B-R	TGAAGCAGCAGGAGCGAATGG
IDO1	IDO1-F	TCATCTCACAGACCACAAGTCACAG
	IDO1-R	TTGGCAAGACCTTACGGACATCTC
SLCO5A1	SLCO5A1-F	GGTGGTGGTGGTGTTCGTCAG
	SLCO5A1-R	GGGCGAGATGAAGTGAGGTAAGG
TNFAIP6	TNFAIP6-F	GCCATCTCGCAACTTACAAGCAG
	TNFAIP6-R	CCCTTAGCCATCCATCCAGCAG
IL6	IL6-F	TGGTGTTGCCTGCTGCCTTC
	IL6-R	GCTGAGATGCCGTCGAGGATG
LAMP3	LAMP3-F	AACAACCGATGTCCAACTTCAAGC
	LAMP3-R	AAGGCAGAGACCAACCACGATG
CCL4L2	CCL4L2-F	TCTCTAGCACTCTCAGCACCAATG
	CCL4L2-R	CTACCACAAAGTTGCGAGGAAGC
OR11L1	OR11L1-F	AGCCCCAAAATACCTCCACTGTG
	OR11L1-R	CCACGGTGATGATGACAACATTCC
HS6ST3	HS6ST3-F	GTGGCTCTTCTCCCGCTTCTC
	HS6ST3-R	TTCCTGGTGTGGCTGTGGTTG
ALOX12	ALOX12-F	CTGGCTCCTGGCAAAGTCCTG

	ALOX12-R	ATGGTGGCGACAGCGATGAC
GOLGA8S	GOLGA8S-F	CCAGCAGGAGCCAAGAGGAAC
	GOLGA8S-R	CTTCAGGGTAGCAGATGATGTAGGG
SH3BGR	SH3BGR-F	AGGCTGAAGAAGGTGGAGAAACTG
	SH3BGR-R	CTTCCGTCTCTTCTGTGGCTGTC
MSTRG.8388.46	MSTRG.8388.46-F	CGCACTAAGTTCGGCATCAATATGG
	MSTRG.8388.46-R	CCTGCTCCGTTTCCGACCTG
MSTRG.24688.13	MSTRG.24688.13-F	CATTGATCGCCAGGGTTGATTCG
	MSTRG.24688.13-R	CTTCGGGAGGGACGCACATG
MSTRG.28053.35	MSTRG.28053.35-F	AGGAGAATCACTTGAACCAGGAAGG
	MSTRG.28053.35-R	CACTCGTCACCCAGGCTAGAATAC
MSTRG.8388.37	MSTRG.8388.37-F	AGCCTGAGCAACATAGCGAGAC
	MSTRG.8388.37-R	AGGTCACCATATTGATGCCGAACTC
MSTRG.17628.2	MSTRG.17628.2-F	CTGCGTGCTGTATCGGAATTGC
	MSTRG.17628.2-R	TTACACCCTTTCTCTGCCAGTATCG
MSTRG.8388.12	MSTRG.8388.12-F	AGCCTGAGCAACATAGCGAGAC
	MSTRG.8388.12-R	CTACAGCCCAGAACTCCTGAACTC
MSTRG.18432.31	MSTRG.18432.31-F	ACTCTGTCGCTAAGGCTGGAATG
	MSTRG.18432.31-R	AGGAGACTGAGGCACAAGAATCAC
MSTRG.21333.4	MSTRG.21333.4-F	GCAGTTGACCTTGTGTGTAGAATGG
	MSTRG.21333.4-R	GACCAGCGGAACAGAGCAGAG
MSTRG.20080.28	MSTRG.20080.28-F	ATTTGACTTGACCTCCCACCAGAC
	MSTRG.20080.28-R	CACACCATCCACAGCATCCATTAAG
MSTRG.8388.13	MSTRG.8388.13-F	CCGTGTTAGCCAGGATGGTCTC
	MSTRG.8388.13-R	ACCTCAGCCTCCCGAGTAGC
MSTRG.29536.4	MSTRG.29536.4-F	AGTACCCAAACTGTGTATTATTATATAGTG
	MSTRG.29536.4-R	ATTTGATGTACCTTCATATTTTATGGACAG

MSTRG.3587.21	MSTRG.3587.21-F	ATAGGAGAACAACACAGCAGTAGGG
	MSTRG.3587.21-R	CAATGGCAAGAGCACACCTGAAC
MSTRG.23498.43	MSTRG.23498.43-F	GTGTTGGGAGATGGGCTGATAGG
	MSTRG.23498.43-R	TCACCACCAGTCATCCAGTCATTC
MSTRG.8385.23	MSTRG.8385.23-F	CCTGGGCAACATAGCGAGACC
	MSTRG.8385.23-R	ATAGAGCAGTGAGGGAGGAAGGG
circ_001974	circ_001974-F	AAGTCAGGCGTGGAATGAAG
	circ_001974-R	GAGCTCTCTGACTACACTGAAAC
circ_040757	circ_040757-F	TGCTGCCTTAACTTACATGCC
	circ_040757-R	ACAACCATTCCTCCACAACC
circ_011057	circ_011057-F	TGCAGCTGTATGCCAGTATG
	circ_011057-R	TCCTTAGCCAAGTAATCTGCAAG
circ_020438	circ_020438-F	TTCCCAAGCACAGCAAAGAG
	circ_020438-R	TCAAGAATCACCACAGGAGAGG
circ_037220	circ_037220-F	TGCCTCCTCTAGAAGCATTGG
	circ_037220-R	AGTCATCTGCTCCATTGTCCAC
circ_008639	circ_008639-F	ATTGTTCTGGGTTGGCATGG
	circ_008639-R	TTTGGCTCCTGGTGTCATTG
circ_043435	circ_043435-F	CACTCTATGGCCTTGGAGAAC
	circ_043435-R	GAACTTTCTTGCCATTCACTGAC
circ_014239	circ_014239-F	GTTCGCCTGTGTTCCTTGATAC
	circ_014239-R	ACTGCTGCGTAGATGGAAGTC
circ_023961	circ_023961-F	TCTTCTCAGCTTGCTCCAAATG
	circ_023961-R	AGCAATCTCCTCATCAAGAAAGC
circ_033309	circ_033309-F	GGCCTTCTGAAATGTGAGCTG
	circ_033309-R	TGTTGAGTCCTTCTGCTTCAGG
circ_005285	circ_005285-F	TTGTCCTTCCCGTTCTCCAG

	circ_005285-R	GTATGACCAGTCCGCCTACG
miR-365a-5p	miR-365a-5p-RT	GTCGTATCCAGTGCAGGGTCCGAGGTATTCGCACT GGATACGACCACATC
	miR-365a-5p-F	AGGGACTTTTGGGGGCA
miR-3190-3p	miR-3190-3p-RT	GTCGTATCCAGTGCAGGGTCCGAGGTATTCGCACT GGATACGACTCTCTG
	miR-3190-3p-F	CGTGTGGAAGGTAGACGGC
miR-1248	miR-1248-RT	GTCGTATCCAGTGCAGGGTCCGAGGTATTCGCACT GGATACGACTTTAGC
	miR-1248-F	GCGACCTTCTTGTATAAGCACTGT
miR-411-5p	miR-411-5p-RT	GTCGTATCCAGTGCAGGGTCCGAGGTATTCGCACT GGATACGACCGTACG
	miR-411-5p-F	CGCGCGTAGTAGACCGTATAG
miR-31-5p	miR-31-5p-RT	GTCGTATCCAGTGCAGGGTCCGAGGTATTCGCACT GGATACGACAGCTAT
	miR-31-5p-F	GCGAGGCAAGATGCTGGC
miR-493-3p	miR-493-3p-RT	GTCGTATCCAGTGCAGGGTCCGAGGTATTCGCACT GGATACGACCCTGGC
	miR-493-3p-F	GCGCGTGAAGGTCTACTGTGT
miR-409-3p	miR-409-3p-RT	GTCGTATCCAGTGCAGGGTCCGAGGTATTCGCACT GGATACGACAGGGGT
	miR-409-3p-F	CGGAATGTTGCTCGGTGA
miR-323a-3p	miR-323a-3p-RT	GTCGTATCCAGTGCAGGGTCCGAGGTATTCGCACT GGATACGACAGAGGT
	miR-323a-3p-F	GCGCACATTACACGGTCG
miR-654-3p	miR-654-3p-RT	GTCGTATCCAGTGCAGGGTCCGAGGTATTCGCACT GGATACGACAAGGTG
	miR-654-3p-F	CGCGTATGTCTGCTGACCAT
miR-370-3p	miR-370-3p-RT	GTCGTATCCAGTGCAGGGTCCGAGGTATTCGCACT GGATACGACACCAGG
	miR-370-3p-F	GGCCTGCTGGGGTGGAA
miR-494-3p	miR-494-3p-RT	GTCGTATCCAGTGCAGGGTCCGAGGTATTCGCACT

		GGATACGACGAGGTT
	miR-494-3p-F	CGCGTGAAACATACACGGGA
miR-127-3p	miR-127-3p-RT	GTCGTATCCAGTGCAGGGTCCGAGGTATTCGCACT GGATACGACAGCCAA
	miR-127-3p-F	CGTCGGATCCGTCTGAGC
miR-381-3p	miR-381-3p-RT	GTCGTATCCAGTGCAGGGTCCGAGGTATTCGCACT GGATACGACACAGAG
	miR-381-3p-F	CGCGTATACAAGGGCAAGCT
P2RX7	P2RX7-F	GCATCACCACCTCAGAGCTGTTC
	P2RX7-R	GCTGGGCAGGATGGCAAAGTC
MALAT1	MALAT1-F	GCAGAGCAAAGGAAGTGGCTTAATG
	MALAT1-R	CATACTGCCAGGCTGGTTATGACTC
miR-20b-5p	miR-20b-5p-RT	CTCGTATCCAGTGCAGGGTCCGAGGTATTCGCACT GGATACGACCTACCT
	miR-20b-5p-F	GCGCAAAGTGCTCATAGTGC
U6	U6-F	CTCGCTTCGGCAGCACA
	U6-R	AACGCTTCACGAATTTGCGT
GAPDH	GAPDH-F	GTCTCCTCTGACTTCCAACAGC
	GAPDH-G	ACCACCCTGTTGCTGTAGCCA

Table S2. Primers used for plasmid construction

Target genes	Primer names	Sequence (5′-3′)
P2RX7	P2RX7-F	GGACTCAGATCTCGAGATGCCGGCCTGCTGCAG
	P2RX7-R	CGGCCGGTGGATCCCGGGTCAGTAAGGACTCTTGAAGCC
MALAT1	MALAT1-F	GGACTCAGATCTCGAGGGCAAATATTGGCAATTAGTTGGC
	MALAT1-R	GATCCGGTGGATCCCGGGTTTATTATTTTGAATGATTTAATGG

Table S3. siRNA sequences

Name	Primer names	siRNA sequence (5′-3′)
Negative control	Negative control-S	UUCUCCGAACGUGUCACGUTT
	Negative control-AS	ACGUGACACGUUCGGAGAATT
si-MALAT1-1#	si-MALAT1-357-S	GGGCUGACAUUAACUACAATT
	si-MALAT1-357-AS	UUGUAGUUAAUGUCAGCCCTT
si-MALAT1-2#	si-MALAT1-1087-S	GGCAGCUGUUAACAGAUAATT
	si-MALAT1-1087-AS	UUAUCUGUUAACAGCUGCCTT
si-MALAT1-3#	si-MALAT1-1312-S	GGCAUUUGCAUCUUUAAAUTT
	si-MALAT1-1312-AS	AUUUAAAGAUGCAAAUGCCTT
si-P2RX7-1#	si-P2RX7-366-S	GCAGACUACACCUUCCCUUTT
	si-P2RX7-366-AS	AAGGGAAGGUGUAGUCUGCTT
si-P2RX7-2#	si-P2RX7-800-S	CCGAGAAACAGGCGAUAAUTT
	si-P2RX7-800-AS	AUUAUCGCCUGUUUCUCGGTT
si-P2RX7-3#	si-P2RX7-1392-S	GCGAUGGACUUCACAGAUUTT
	si-P2RX7-1392-AS	AAUCUGUGAAGUCCAUCGCTT
si-P2RX7-4#	si-P2RX7-1819-S	GGAGGAUCCGGAAAGAGUUTT
	si-P2RX7-1819-AS	AACUCUUUCCGGAUCCUCCTT
miRNA-inhibitor NC	miRNA-inhibitor NC	CAGUACUUUUGUGUAGUACAA
miR-20b-5p mimic	miR-20b-5p mimic-S	CAAAGUGCUCUAUAGUGCAGGCAG
	miR-20b-5p mimic-AS	ACCUGCACUAUGAGCACUUUGUU
miR-20b-5p inhibitor	miR-20b-5p inhibitor-S	CUACCUGCACUAUGAAGCACUUUG

Table S4. Oligonucleotide sequences for dual-luciferase reporter assay

Gene	Type	Sequence (5′-3′)
MALAT1	WT	GATAAGTTTAACTTGCATCTGCAGTATTGCATGTTAGGGATAAG TGCTTATTTTTAAGAGCTGTGGAGTTCTTAAATATCAACCATGG CACTTTCTCCTGACCCCTTCCCTAGGGGATTTCAGGATTGAGA AATTTTTCCATCGAGCCTTTTTAAAATTGTAGGACTTGTTCCTGT GGGCTTCAGTGATGGGATAGTACA
MALAT1	MUT	GATAAGTTTAACTTGCATCTGCAGTATTGCATGTTAGGGATAAG TGCTTATTTTTAAGAGCTGTGGAGTTCTTAAATATCAACCTACC GTGAAACTCCTGACCCCTTCCCTAGGGGATTTCAGGATTGAGA AATTTTTCCATCGAGCCTTTTTAAAATTGTAGGACTTGTTCCTGT GGGCTTCAGTGATGGGATAGTACA
P2RX7	WT	TGGAACTCTTGCTATCGGGGAAGCCAGACGCCATTTAAAAGTC TGCCTATCCTGGCCAGGTGTGGTGGCTCACACCTGTAATCCCA GCACTTTGGGAGACCAAGGCGGGCGGATCACTTAAAGTCAGG AGTCCAAGACCAGACTCGCCAACATGGTGAAACCGTATCTCT AATAAAAATACAAAAATTAGCTGGGCATGG
P2RX7	MUT	TGGAACTCTTGCTATCGGGGAAGCCAGACGCCATTTAAAAGTC TGCCTATCCTGGCCAGGTGTGGTGGCTCACACCTGTAATCCCA CGTGAAAGGGAGACCAAGGCGGGCGGATCACTTAAAGTCAG GAGTCCAAGACCAGACTCGCCAACATGGTGAAACCGTATCTC TAATAAAAATACAAAAATTAGCTGGGCATGG

Table S5. Differentially expressed mRNAs, lncRNAs, miRNAs, and circRNAs

Number	Name	$\log_2$ (Fold Change)	q-Value
1	TSLP	3.9881	8.537E-28
2	CXCL1	3.7294	3.099E-57
3	CXCL8	3.3846	0
4	CXCL2	3.2020	4.084E-29
5	BIRC3	3.1478	0
6	SOD2	2.8865	0
7	OLR1	2.4851	1.299E-230
8	IL1B	2.4576	0
9	GBP2	2.2551	3.337E-180
10	P2RX7	2.1380	1.733E-31
11	MEF2C-AS1	5.2706	0.0060
12	IQCH-AS1	3.8569	0.0017
13	GOT1-DT	3.6508	0.0184
14	METTL14-DT	3.4304	0.0320
15	FTX	3.2426	0.0486
16	RPAP3-DT	3.1075	0.0388
17	ENSG00000286285	2.3459	0.0348
18	SNHG5	2.1398	0.0208
19	MALAT1	2.0107	0.0151
20	ZNF225-AS1	1.9778	0.0316
21	miR-206	-6.9505	0.0120
22	miR-205-5p	-6.1896	3.917E-0X8
23	miR-379-5p	-4.5394	1.128E-15
24	miR-494-3p	-4.4898	0.0006
25	miR-370-3p	-4.2628	6.905E-15

26	miR-409-3p	-3.8718	5.512E-09
27	miR-31-5p	-3.2276	0.0001
28	miR-134-5p	-2.6879	0.0037
29	miR-20b-5p	-1.7608	0.0014
30	miR-429	-1.4157	0.0103
31	circ_001974	3.6889	0.0447
32	circ_040757	3.6128	0.0334
33	circ_011057	3.2386	0.0048
34	circ_020438	2.6389	0.0478
35	circ_037220	2.4657	0.0179
36	circ_008639	2.0847	0.0198
37	circ_043435	1.8965	0.0244
38	circ_014239	1.7866	0.0223
39	circ_023961	1.3206	0.0362
40	circ_033309	-2.5699	0.0015

Table S6. lncRNAs used for ceRNA network construction

Number	Name	log_2 (Fold Change)	q-Value
1	MSTRG.8388	16.7642	0.0045
2	MSTRG.17628	11.6370	0.0320
3	MSTRG.18432	11.2785	0.0000
4	MSTRG.19704	9.6566	0.0497
5	MSTRG.12364	9.3345	0.0000
6	MSTRG.12674	8.5813	0.0000
7	LINC01282	8.3472	0.0000
8	MSTRG.4369	6.6113	0.0016
9	MSTRG.17151	6.1771	0.0022
10	MSTRG.23373	6.1510	0.0080
11	MSTRG.19954	5.8707	0.0289
12	MSTRG.1033	5.8684	0.0021
13	MSTRG.780	5.6926	0.0023
14	LINC01140	5.3945	0.0308
15	MEF2C-AS1	5.2706	0.0060
16	MSTRG.17151	5.2442	0.0113
17	ENSG00000290531	5.0188	0.0119
18	MIR9-1HG	4.9988	0.0001
19	CASC15	4.9084	0.0268
20	MSTRG.1879	4.8356	0.0404
21	PCBP1-AS1	4.7758	0.0352
22	MSTRG.21336	4.7349	0.0462
23	MSTRG.5394	4.6444	0.0465
24	MSTRG.16340	4.5792	0.0128
25	LINC00667	4.5072	0.0155

26	PCBP1-AS1	4.4098	0.0261
27	MSTRG.25077	4.3264	0.0001
28	MSTRG.887	4.2698	0.0471
29	ENSG00000291081	4.2081	0.0007
30	ENSG00000230647	4.1202	0.0002
31	MSTRG.27643	4.1003	0.0171
32	LINC02757	4.0585	0.0374
33	LINC00926	3.9968	0.0187
34	LINC01539	3.9310	0.0391
35	ENSG00000286695	3.8729	0.0231
36	IQCH-AS1	3.8569	0.0017
37	ENSG00000239775	3.8304	0.0013
38	ENSG00000287917	3.7094	0.0048
39	GOT1-DT	3.6508	0.0185
40	ENSG00000289914	3.6066	0.0095
41	MSTRG.28334	3.5812	0.0005
42	ENSG00000289198	3.5634	0.0016
43	HMGA2-AS1	3.5242	0.0064
44	METTL14-DT	3.4304	0.0320
45	ENSG00000289589	3.3648	0.0015
46	MSTRG.23774	3.3262	0.0001
47	ENSG00000289485	3.2575	0.0262
48	FTX	3.2426	0.0486
49	MSTRG.4337	3.2404	0.0002
50	LINC00852	3.2392	0.0376
51	MSTRG.21588	3.2200	0.0403
52	MSTRG.23498	3.2149	0.0422

53	ENSG00000290933	3.2057	0.0469
54	IQCH-AS1	3.1988	0.0423
55	RAD51-AS1	3.1689	0.0254
56	RAD51-AS1	3.1689	0.0254
57	RPAP3-DT	3.1075	0.0388
58	MSTRG.27940	3.0765	0.0069
59	MSTRG.28907	2.9687	0.0005
60	MSTRG.5177	2.9413	0.0060
61	LINC02561	2.9191	0.0325
62	MSTRG.5389	2.9029	0.0012
63	MSTRG.9067	2.8831	0.0004
64	ENSG00000289470	2.7541	0.0084
65	MSTRG.11135	2.7391	0.0018
66	MSTRG.1643	2.7235	0.0009
67	MSTRG.14123	2.6640	0.0017
68	ENSG00000287216	2.5844	0.0054
69	MSTRG.27643	2.5061	0.0226
70	DENND3-AS1	2.4943	0.0187
71	MSTRG.2100	2.4892	0.0039
72	ENSG00000290041	2.4575	0.0454
73	LINC03014	2.4454	0.0296
74	MSTRG.21926	2.4110	0.0022
75	MSTRG.2539	2.4044	0.0335
76	LINC01894	2.3957	0.0224
77	ENSG00000286285	2.3459	0.0348
78	MSTRG.6716	2.3450	0.0065
79	WAKMAR2	2.2985	0.0278

80	ENSG00000257027	2.2654	0.0149
81	SNHG16	2.2625	0.0050
82	MSTRG.12322	2.2526	0.0219
83	MSTRG.19005	2.2163	0.0070
84	MSTRG.28587	2.2090	0.0066
85	MSTRG.26560	2.1477	0.0267
86	SNHG5	2.1398	0.0208
87	MALAT1	2.0107	0.0151
88	SNHG29	1.9974	0.0125
89	ZNF225-AS1	1.9778	0.0316
90	FTX	1.9264	0.0307
91	MSTRG.18259	1.9193	0.0270
92	MSTRG.3520	1.9015	0.0203
93	LINC01191	1.8898	0.0218
94	MSTRG.24745	1.8847	0.0305
95	MSTRG.21774	1.8787	0.0456
96	LINC02562	1.8650	0.0436
97	MSTRG.50	1.7947	0.0219
98	ENSG00000291220	1.7341	0.0454
99	SNHG29	1.5622	0.0470
100	MSTRG.4973	-1.7127	0.0277
101	ENSG00000258168	-1.9867	0.0176
102	PAX8-AS1	-2.0444	0.0492
103	ENSG00000278467	-2.0647	0.0344
104	BMS1P1	-2.1797	0.0158
105	ATP6V0D1-DT	-2.2533	0.0230
106	MSTRG.4337	-2.2697	0.0049

107	ENSG00000279679	-2.2700	0.0048
108	MSTRG.8555	-2.3592	0.0063
109	MSTRG.5191	-2.4075	0.0035
110	SNHG5	-3.0621	0.0269
111	TTC28-AS1	-3.0770	0.0240
112	MSTRG.18248	-3.3290	0.0069
113	TMEM161B-DT	-3.4971	0.0173
114	MSTRG.26	-3.7954	0.0124
115	MCPH1-AS1	-5.7694	0.0082
116	ENSG00000291144	-7.1625	0.0000
117	MSTRG.322	-7.2582	0.0000
118	MSTRG.23498	-14.0558	0.0096

Table S7. miRNAs used for ceRNA network construction

Number	Name	$\log_2$ (Fold Change)	q-Value
1	miR-1248	1.0420	0.0037
2	miR-3190-3p	1.1671	0.0442
3	miR-206	-6.9505	0.0120
4	miR-205-5p	-6.1896	0.0000
5	miR-381-3p	-5.4664	0.0000
6	miR-379-5p	-4.5394	0.0000
7	miR-494-3p	-4.4898	0.0006
8	miR-409-5p	-4.3643	0.0024
9	miR-370-3p	-4.2628	0.0000
10	miR-493-5p	-4.2435	0.0000
11	miR-654-3p	-4.1439	0.0000
12	miR-323a-3p	-4.0514	0.0066
13	miR-409-3p	-3.8718	0.0000
14	miR-379-3p	-3.5614	0.0362
15	miR-483-3p	-3.5478	0.0366
16	miR-493-3p	-3.4660	0.0000
17	miR-31-5p	-3.2276	0.0001
18	miR-411-5p	-3.0512	0.0000
19	miR-134-5p	-2.6879	0.0037
20	miR-20b-5p	-1.7608	0.0014
21	let-7c-5p	-1.4571	0.0000
22	miR-429	-1.4157	0.0103

Table S8. mRNAs used for ceRNA network construction

Number	Name	log_2 (Fold Change)	q-Value
1	TNFAIP6	4.8920	4.158E-62
2	VCAM1	4.1677	1.194E-13
3	CSF2	4.0729	1.071E-17
4	CD80	4.0205	1.24E-150
5	TSLP	3.9881	8.537E-28
6	ACOD1	3.7653	4.462E-08
7	CXCL1	3.7294	3.099E-57
8	RNF144B	3.6731	1.385E-73
9	CXCL8	3.3846	0
10	CD274	3.2718	8.975E-22
11	CCL3	3.2169	1.15E-239
12	CXCL2	3.2020	4.084E-29
13	EVA1A	3.2005	9.993E-43
14	BIRC3	3.1478	0
15	MARCKS	2.9464	0
16	CXCL3	2.9189	3.509E-33
17	PSTPIP2	2.9167	6.64E-139
18	SOD2	2.8865	0
19	KCNA3	2.7881	1.275E-40
20	IL1A	2.6806	6.095E-15
21	CCL22	2.6540	3.579E-58
22	IRAK2	2.5009	1.34E-211
23	ADORA2A	2.5001	2.123E-17
24	OLR1	2.4851	1.3E-230
25	SOCS3	2.4831	1.032E-23

26	IL10	2.4701	0.0084895
27	PTGS2	2.4605	1.39E-97
28	IL1B	2.4576	0
29	SERPINB2	2.4253	6.123E-08
30	SRC	2.4052	6.56E-127
31	IER3	2.3304	0
32	CCL20	2.2675	3.62E-90
33	GBP2	2.2551	3.34E-180
34	FGF2	2.2513	2.07E-173
35	IL33	2.2396	3.544E-05
36	TNFRSF18	2.2326	1.638E-12
37	SIK1	2.1712	0.0238888
38	P2RX7	2.1380	1.733E-31
39	CCR7	2.0816	1.152E-06
40	DRAM1	2.0110	2.611E-63
41	NRG1	2.0030	6E-305
42	TNFSF11	-2.0276	0.0017981
43	CXCR2	-2.1765	1.14E-06
44	PDK4	-2.1881	6.706E-15
45	MERTK	-2.2848	0
46	FGD4	-2.4453	9.477E-76
47	GJA1	-4.4801	0.0082131
